# Rocaglamide Suppresses Allergic Reactions by Regulating IL-4 Receptor Signaling

**DOI:** 10.3390/molecules30040840

**Published:** 2025-02-11

**Authors:** Hyein Jo, Misun Kim, Jaewhoon Jeoung, Wonho Kim, Yoon Ho Park, Hyun Suk Jung, Wook Lee, Dooil Jeoung

**Affiliations:** Department of Biochemistry, Kangwon National University, Chuncheon 24341, Republic of Korea; qnfdudn1212@gmail.com (H.J.); misun210@gmail.com (M.K.); heyjhw@kangwon.ac.kr (J.J.); kimwonho99@kangwon.ac.kr (W.K.); hsjung@kangwon.ac.kr (H.S.J.); wlee@kangwon.ac.kr (W.L.)

**Keywords:** allergic reactions, interleukin-4 receptor, miR-34a, molecular docking, Rocaglamide

## Abstract

Rocaglamide (Roc-A), a natural phytochemical isolated from Aglaia species, is known to exert anticancer effects. Allergic inflammation can enhance the tumorigenic potential of cancer cells. We hypothesized that Roc-A could regulate allergic inflammation. Roc-A prevented an antigen from increasing the hallmarks of allergic reactions in vitro. Roc-A suppressed passive cutaneous anaphylaxis (PCA) and passive systemic anaphylaxis (PSA). RNA sequencing analysis showed that Roc-A prevented the antigen from increasing the expression of IL-4 in RBL2H3 cells. Roc-A also prevented the antigen from increasing the expression of interleukin-4 receptor (IL-4R). Roc-A was found to form a hydrogen-bonding network with residues N92 and L64 of IL-4R in a molecular docking simulation. Roc-A prevented the antigen from inducing the binding of IL-4R to JAK1. Chromatin immunoprecipitation (ChIP) assays showed that C-Jun could bind to promoter sequences of IL-4 and IL-4R. Mouse recombinant IL-4 protein increased β-hexosaminidase activity, IL-4R expression, and the hallmarks of allergic inflammation in the antigen-independent manner. Mouse recombinant IL-4 protein increased the expressions of CD163 and arghinase-1 and markers of M2 macrophages, but decreased the expression of iNOS, a marker of M1 macrophages in lung macrophages. Roc-A regulated the effects of a culture medium of antigen-stimulated RBL2H3 cells on the expressions of iNOS and arginase-1 in RAW264.7 macrophages. The blocking of IL-4 or downregulation of IL-4R exerted negative effects on the hallmarks of allergic reactions in vitro. The blocking of IL-4 or downregulation of IL-4R also exerted negative effects on PCA, and the downregulation of IL-4R exerted negative effects on PSA. An miR-34a mimic exerted negative effects on allergic reactions in vitro. The downregulation of IL-4R prevented the antigen from decreasing the expression of miR-34a in RBL2H3 cells. We identified chemicals that could bind to IL-4R via molecular docking analysis. The IL-4R docking chemical 1536801 prevented the antigen from increasing β-hexosaminidase activity and the hallmarks of allergic reactions. The IL-4R docking chemical 1536801 also exerted a negative effect on PCA. TargetScan analysis predicted miR-34a as a negative regulator of IL-4R. We found that the anti-allergic effect of Roc-A and its mechanisms were associated with miR-34a. Taken together, our results show that understanding IL-4R-mediated allergic reactions can provide clues for the development of anti-allergy therapeutics.

## 1. Introduction

Rocaglamide (Roc-A) inhibits translation initiation and elongation by clamping eukaryotic initiation factor 4A (eIF4A) to poly-purine sequences in coding regions [1]. The expression of eIF4A is upregulated in invasive breast cancer [2]. This suggests that Roc-A possesses an anticancer effect. Roc-A can suppress the self-renewal ability of breast cancer stem cells by decreasing the expressions of Nanog homeobox (NANOG) and Octamer binding protein-4 (OCT4) [3]. Roc-A can also suppress the metastasis of pancreatic cancer cells by targeting eIF4 [4]. In addition, Roc-A can inhibit epidermal growth factor (EGF)/RAS-induced c-RAF kinase activation by targeting prohibitin (PHB1) in lung tumors [5]. Roc-A can also decrease the expression of c-MYC in chronic lymphocytic leukemia cells [6] while inducing caspase-mediated apoptosis by exerting the depolarization of the mitochondrial membrane potential [7]. Roc-A can induce apoptosis through the intrinsic death pathway in various human leukemia cell lines [7]. Roc-A can also enhance the sensitivity of TNF-related apoptosis, inducing ligand (TRAIL)-resistant renal cell carcinoma cells to TRAIL by decreasing the expression of c-FLICE-like inhibitory protein (c-FLIP) [8].

Allergic inflammation is known to enhance the tumorigenic potential of cancer cells by inducing cellular interactions [9,10]. LIM and SH3 protein 1 (LASP1) and CXCR4 are critical for eIF4A to translate oncogenic mRNAs into oncoproteins [2]. CXCR4 signaling is necessary for interactions between fibroblasts and immune cells during allergic airway inflammation [11]. It is known that Roc-A can regulate the expression of eIF4A. Since Roc-A inhibits tumorigenic potential, it might affect cellular interactions involving cancer cells, macrophages, and mast cells during allergic inflammations. Roc-A can suppress CD4^+^ T cell activation [12]. These reports suggest a possible role of Roc-A in allergic reactions.

Allergic inflammations are accompanied by an increased number of active Th2 cells [13,14]. Peanut allergy remission is accompanied by a reduction in Th2 activity [15]. IL-4 and IL-5, Th2 cytokines that are targets of secretoglobin family 1C member 1 (SCGB1C1), can mediate allergic airway inflammation [16]. Cancer-cell-derived IL-4 can induce M2-like polarization in pancreatic cancer cells [17]. The M2 polarization of macrophages contributes to the pathogenesis of anaphylaxis [9,10]. This implies a role of IL-4 signaling in the M2 polarization of macrophages during allergic inflammation. These reports imply that Roc-A might target IL-4 signaling to exert anti-allergic effects, if any.

This study aimed to examine the effects of Roc-A on allergic reactions. Roc-A was found to suppress allergic reactions both in vitro and in vivo. RNA sequencing analysis suggested that the IL-4 signaling axis might serve as a target of Roc-A. We examined the mechanism involved in the expression regulation of IL-4 and IL-4R by Roc-A. IL-4 and IL-4R were shown to mediate anaphylaxis. We also found that miR-34, predicted as a negative regulator of IL-4R, negatively regulated allergic reactions in vitro by decreasing the expression of IL-4R. Molecular docking analysis showed the potential binding of Roc-A to IL-4R. We identified chemicals that could bind to IL-4R by employing molecular docking analysis. Among the identified chemicals, chemical 1536801 was shown to inhibit allergic reactions both in vitro and in vivo. This study presents evidence that IL-4R signaling can be a target for developing anti-allergy therapeutics.

## 2. Results

### 2.1. Roc-A Inhibits Allergic Reactions In Vitro

**Figure 1:**
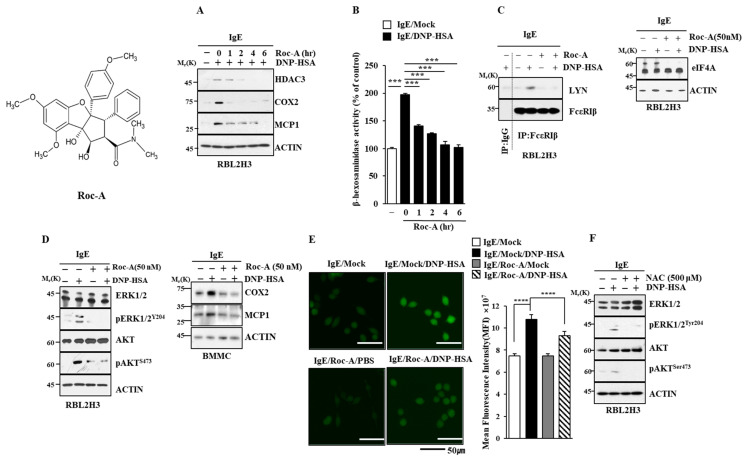
Roc-A inhibits allergic reactions in vitro. (**A**) DNP-specific IgE (100 ng/mL)-sensitized RBL2H3 cells were treated without or with Roc-A (1 μM) for various time intervals, followed by stimulation with DNP-HSA (100 ng/mL) for 1 h. Representative blots are shown. (**B**) β-hexosaminidase activity assays were performed. The means ± S.E. of three independent experiments are shown. One-way ANOVA was carried out. ***, *p* < 0.001. (**C**) DNP-specific IgE (100 ng/mL)-sensitized RBL2H3 cells were treated without or with Roc-A (1 μM) for 2 h. Immunoprecipitation and immunoblot (right) were performed. Immunoprecipitation employing isotype-matched IgG antibody (1 μg/mL) was also performed. (**D**) DNP-specific IgE (100 ng/mL)-sensitized RBL2H3 cells or BMMCs were treated without or with Roc-A for 2 h. (**E**) DCFH-DA (10 μM) was added 20 min after the addition of DNP-HSA (100 ng/mL). ****, *p* < 0.0001. (**F**) DNP-specific IgE (100 ng/mL)-sensitized RBL2H3 cells were treated without or with NAC (500 µM) for 2 h.

Allergic inflammation can promote the tumorigenic and metastatic potential of cancer cells [9,10]. The activation of mast cells contributes to the pathogenesis of carcinogenesis [18]. Since Roc-A, a natural phytochemical isolated from Aglaia species, exerts anticancer activity [5], the effects of Roc-A on allergic reactions were examined. Roc-A exerted negative effects on the increased expression of hallmarks of allergic inflammation such as HDAC3, COX2, and MCP1 in antigen (DNP-HSA)-stimulated RBL2H3 cells (Figure 1A). Monocyte chemoattractant protein1 (MCP1) (CCL2) is a potent histamine-releasing factor [19]. Roc-A prevented the antigen from increasing β-hexosaminidase activity (Figure 1B). Roc-A inhibited the binding of FcεRI to LYN in RBL2H3 cells (Figure 1C). However, Roc-A did not affect the expression of eIF-4, a target of Roc-A (Figure 1C). Roc-A prevented the antigen from increasing the phosphorylation levels of extracellular-regulated kinase (ERK) and AKT in RBL2H3 cells (Figure 1D) and the expression of cyclooxygenase 2 (COX2) and MCP1 in bone-marrow-derived mast cells (Figure 1D). Allergic asthma is characterized by IgE–FcεRI cross linking and AKT activation [20].

Reactive oxygen species (ROS) can mediate vanadium-induced allergic airway inflammation [21]. Antioxidants are known to inhibit allergic airway hyper-responsiveness and decrease house dust mite (HDM)-specific IgE production [22]. Roc-A prevented the antigen from increasing the ROS level in RBL2H3 cells (Figure 1E). The inhibition of ROS formation by N-acetyl L-cysteine (NAC) prevented the antigen from increasing the phosphorylation levels of ERK and AKT (Figure 1F) and β-hexosaminidase activity in RBL2H3 cells (Figure S1A). An AKT inhibitor prevented the antigen from increasing the ROS level (Figure S1B) and hallmarks of allergic inflammation (Figure S1C). ERK and AKT were necessary for increased β-hexosaminidase activity in RBL2H3 cells (Figure S1D,E). Taken together, these results indicate that Roc-A can inhibit allergic reactions in vitro.

### 2.2. Roc-A Inhibits Anaphylaxis

**Figure 2:**
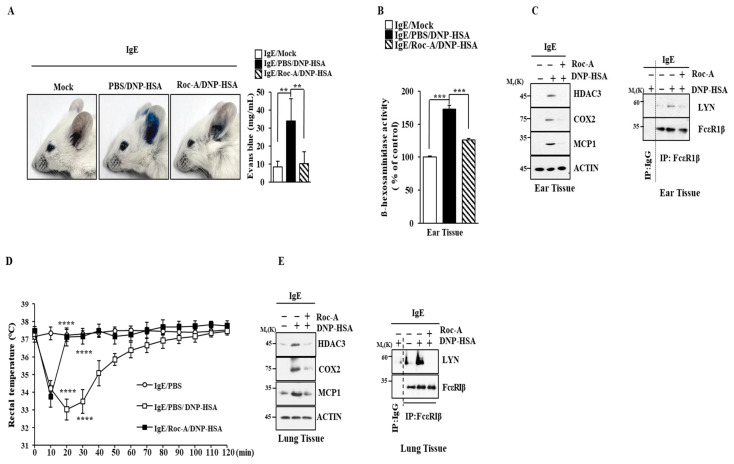
Roc-A inhibits anaphylaxis. (**A**) BALB/C mice were given an intradermal injection of DNP-specific IgE (4 μg/kg) was intradermally injected into the ear and Roc-A (1 μg/kg) was intravenously injected. The next day, intravenous injections of PBS or DNP-HSA (5 mg/kg) and 2% (*v*/*v*) Evans blue solution were performed. Each experimental group comprised four BALB/C mice. **, *p* < 0.01. (**B**) β-hexosaminidase activity assays of ear tissue lysates were performed. ***, *p* < 0.001. (**C**) Immunoblot and immunoprecipitation were performed. (**D**) Intravenous injections with DNP-specific IgE (0.5 mg/kg) and Roc-A (505.567 μg/kg) into BALB/C mice were performed. Twenty-four hours later, DNP-HSA (5 mg/kg) was intravenously injected into BALB/C mice. Rectal temperatures were measured at each time point. ****, *p* < 0.0001. Each experimental group comprised four BALB/C mice. Comparison was made between IgE/DNP-HSA and IgE/Roc-A/DNP-HSA. (**E**) Lung tissue lysates were subjected to immunoblot and immunoprecipitation.

Next, the effects of Roc-A on allergic reactions in vivo were then examined. Roc-A prevented the antigen from enhancing vascular permeability in passive cutaneous anaphylaxis (PCA) (Figure 2A). Roc-A exerted negative effects on the increase in β-hexosaminidase activity (Figure 2B) and hallmarks of allergic inflammation. It inhibited the binding of FcεRI to LYN (Figure 2C). Roc-A prevented the antigen from decreasing the rectal temperature in a mouse model of passive systemic anaphylaxis (PSA) (Figure 2D). Roc-A exerted negative effects on increased hallmarks of allergic inflammation and inhibited the binding of FcεRI to LYN (Figure 2E). The effect of Roc-A on anaphylaxis has not been reported yet.

### 2.3. Roc-A Prevents Antigen from Increasing the Expression of IL-4 and IL-4R

**Figure 3:**
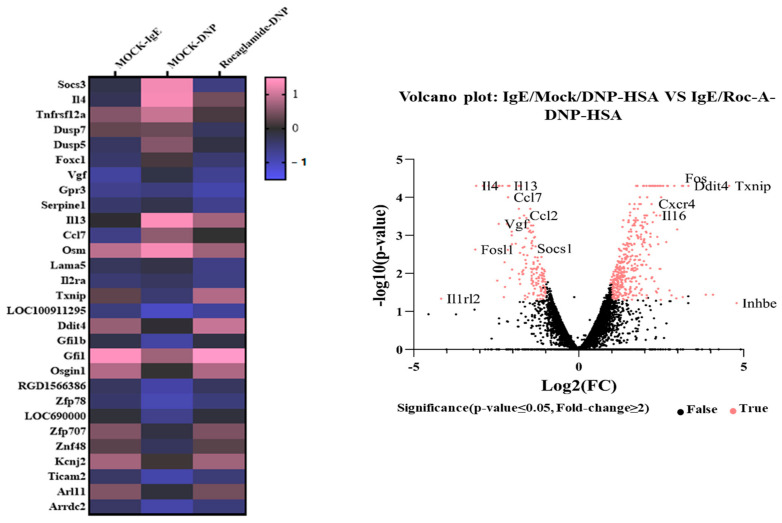
Roc-A prevents antigen from increasing the expression of IL-4. DNP-specific IgE (100 ng/mL)-sensitized RBL2H3 cells were treated without or with Roc-A (1 μM) for 2 h. RNA sequencing analysis was performed. The heat map of expression values of the selected DEGs in log10 (FPKM) units was compared across genes and samples (fold changes > 2 and *p*-value < 0.05).

We identified downstream targets of Roc-A. RNA sequencing analysis showed that IL-3, IL-4, IL-13, suppressor of cytokine signaling 3 (SOCS3), CCL-7, MCP1, forkhead box C1 (FOXC1), and tumor necrosis factor receptor superfamily 12A (TNFRSF12A) were highly increased by antigen stimulation in RBL2H3 cells (Figure 3). The IL-2 family has five members (IL-2, IL-4/13, IL-7, IL-15, and IL-21). Their receptors share a common γc subunit. They have six other subunits (IL-2Rβ1/2, IL-4Rα1/2, IL-13Rα1/2, IL-7Rα, IL-15Rα, and IL-21Rα1/2). IL-3 can induce the anaphylactic degranulation of basophils [23]. Ovalbumin (OVA)-induced allergic asthma is mediated by increased levels of Th2 cytokines [24]. The roles of IL-4 and IL-13 in allergic airway inflammation have been reported [25]. CCL-7 contributes to the pathogenesis of type 2 allergic inflammation induced by IL-33 [26]. RNA sequencing analysis showed that antigen stimulation increased MCP1 expression (Figure 3). MCP1 mediates IgE-induced anaphylaxis [27]. Forkhead box C1 (FOXC1), a transcription factor, can promote the proliferation of airway smooth muscle cells by activating NF-kB in a mouse model of asthma [28]. High levels of TNFRSF12A contribute to the pathogenesis of severe asthma [29]. We focused our study on IL-4, because IL-4 was one of the most highly upregulated genes. In addition, IL-4R signaling could increase the CXCL1 level in a mouse model of atopic dermatitis [30].

**Figure 4:**
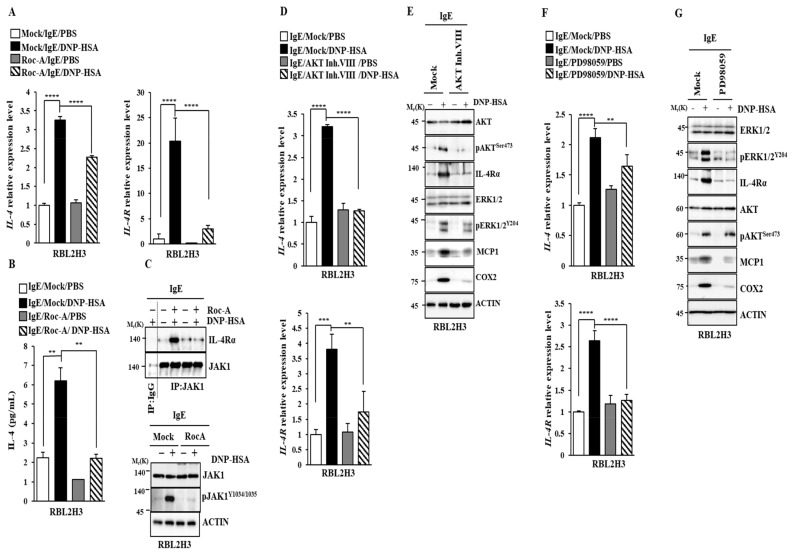
Roc-A, ERK, and AKT regulate the expressions of IL-4 and IL-4R. (**A**) DNP-specific IgE (100 ng/mL)-sensitized RBL2H3 cells were treated without or with Roc-A (1 μM) for 2 h. QRT-PCR was performed. ****, *p* < 0.0001. (**B**) Same as (**A**), except that IL-4 ELISA was performed. **, *p* < 0.01. (**C**) Same as (**B**), except that immunoblot and immunoprecipitation were performed. DNP-specific IgE (100 ng/mL)-sensitized RBL2H3 cells were treated without or with AKT inhibitor VIII (1 μM) for 2 h. QRT-PCR (**D**) or immunoblot (**E**) was performed. **, *p* < 0.01; ***, *p* < 0.001; ****, *p* < 0.0001. (**F**) DNP-specific IgE (100 ng/mL)-sensitized RBL2H3 cells were treated without or with PD98059 (20 μM), an ERK inhibitor, for 2 h. QRT-PCR was performed. **, *p* < 0.01; ****, *p* < 0.0001. (**G**) Same as (**F**), except that immunoblot was performed.

Roc-A decreased the transcriptional levels of IL-4 and IL-4R in antigen-stimulated RBL2H3 cells (Figure 4A). ELISA employing cell lysates showed that Roc-A decreased the IL-4 level (Figure 4B). IL-4R can utilize Janus kinase 1 (JAK1) for signal transduction in food allergies [31]. JAK1-signal transducer and activator of T cells 6 (STAT6) signaling mediates the M2 polarization of macrophages induced by IL-4 [32]. The M2 polarization of macrophages regulated by the miR-154-MCP axis contributes to the pathogenesis of anaphylaxis [33]. Roc-A prevented the antigen from inducing the binding of JAK1 to IL-4R and also from increasing the phosphorylation of JAK1 (Figure 4C). An AKT inhibitor (Figure 4D) and ERK inhibitor (Figure 4F) prevented the antigen from increasing the expression levels of IL-4 and IL-4R at the transcriptional level. Both the AKT inhibitor (Figure 4E) and ERK inhibitor (Figure 4G) prevented the antigen from increasing the expression of IL-4R and hallmarks of allergic reactions. Taken together, these results suggest that Roc-A can inhibit allergic reactions by targeting IL-4 R signaling.

### 2.4. Potential Binding of Rocaglamide to IL-4R by Using In Silico Molecular Docking Analysis

**Figure 5:**
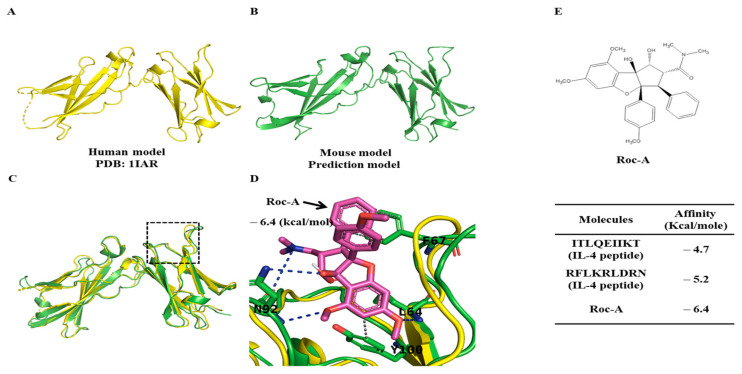
Potential binding of Roc-A to IL-4 R. (**A**) Structure of human IL-4R (colored in yellow; PDB ID: 1IAR). (**B**) Structure of mouse IL-4R (colored in green; produced from Alphafold) and enlarged region of the structure, which were observed near the Roc-A binding site (dotted rectangular region in (**C**)). (**C**) Superimposed image from (**A**) to (**B**). (**D**) The best docking pose of Roc-A (colored in purple) to its binding of the model (the rectangular site in (**C**)), taken from in silico molecular docking analysis. Note that gray and blue dotted lines demonstrate hydrophobic interactions and hydrogen bonds, respectively. (**E**) Table of docking result. Those peptide fragments were extracted from IL-4 structure (PDB ID: 1IAR), which interacted with IL-4R, and were subjected to molecular docking to calculate their binding affinities.

We next examined whether Roc-A could bind to IL-4R. We first examined the structural homology between human and mouse IL-4R by performing sequence alignment and comparing the predicted structure using AphaFold2. The IL-4R human and mouse sequences had an identity of 52.87% and a similarity of 64.48% (Vector Builder. 2023). The structural similarity between the crystal structure of human IL-4R (PDB ID: 1IAR) and AlphaFold2 model of mouse IL-4R is shown in their superimposed structures (Figure 5C). It appeared that the average discrepancy between these models was approximately two angstroms, demonstrating that these two structures exhibited a satisfactory level of correspondence. Thus, we used the prediction model of mouse IL-4R for further analysis and conducted a docking simulation with Roc-A (Figure 5D). Based on the outcomes of the docking simulation, the docking pose with the lowest calculated affinity (−6.4 Kcal/mole) was selected as the most optimal docking pose and its interactions were analyzed (Figure 5D). It was found that IL-4R residues (L64, F67, N92, and Y100) were involved in interactions with Roc-A. Specifically, the L64, F67, and Y100 residues of IL-4R were implicated in hydrophobic interactions, while the L64 and N92 residues of IL-4R were implicated in hydrogen bonding (Figure 5D).

To validate the binding of Roc-A to IL-4R, we conducted docking simulations utilizing interaction residues extracted from the crystal structure of the IL-4/IL-4R complex (PDB ID: 1IAR). Small peptide fragments corresponding to these interaction residues were subjected to docking simulations, employing the same methodology as that for Roc-A. The results of these docking simulations were subsequently compared (Figure 5E). Notably, the calculated affinities for these peptide fragments were lower than those obtained for Roc-A, indicating that the docking pose of Roc-A was stable and favorable for binding to IL-4R. These findings provide compelling evidence for the potential of Roc-A to directly interact with IL-4R to inhibit allergic reactions.

### 2.5. C-Jun Directly Regulates the Expression of IL-4 and IL-4R

**Figure 6:**
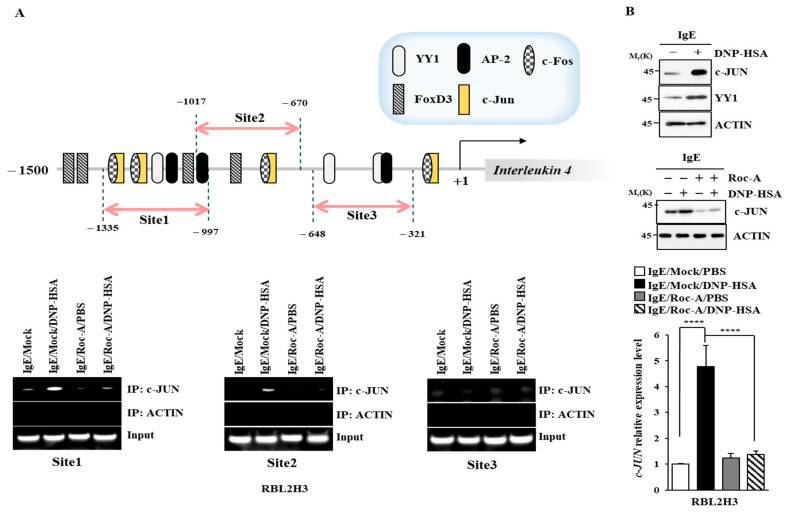
C-Jun directly regulates the expression of IL-4. (**A**) Upper panel shows binding sites for transcription factors in the promoter sequences of IL-4. DNP-specific IgE (100 ng/mL)-sensitized RBL2H3 cells were treated without or with Roc-A (1 μM) for 2 h. ChIP assays were performed (lower). (**B**) DNP-specific IgE (100 ng/mL)-sensitized RBL2H3 cells were stimulated with DNP-HSA (100 ng/mL) for 1 h (upper). DNP-specific IgE (100 ng/mL)-sensitized RBL2H3 cells were treated without or with Roc-A (1 μM) for 2 h, followed by stimulation with DNP-HSA (100 ng/mL). Immunoblot (middle) and qRT-PCR (lower) were performed. ****, *p* < 0.0001.

We examined the mechanisms involved in the upregulation of IL-4. IL-4 promoter sequences contain putative binding sites for YY1, activator prptein-2 (AP-2), C-Jun, forkhead box D3 (FOXD3), and C-Fos (Figure 6A). An increased expression of C-Jun mediates allergen-induced mast cell degranulation [34]. Chromatin immunoprecipitation (ChIP) assays showed that C-Jun could bind to these promoter sequences of IL-4 (Figure 6A). Roc-A prevented the antigen from increasing the expression of C-Jun and from increasing the transcriptional level of C-Jun (Figure 6B). IL-4R promoter sequences contain putative binding sites for YY1, C-Fos, C-Jun, and FOXD3 (Figure S2A). C-Jun could bind to these promoter sequences of IL-4R (Figure S2A). Thus, C-Jun can directly increase the expressions of IL-4 and IL-4R. The downregulation of C-Jun prevented the antigen from increasing the β-hexosaminidase activity in RBL2H3 cells (Figure S2B). C-Jun was necessary for increased transcriptional levels of IL-4 and IL-4R in RBL2H3 cells (Figure S2C). The downregulation of C-Jun prevented the antigen from increasing the expression of IL-4R and hallmarks of allergic inflammation (Figure S2D).

### 2.6. IL-4 and IL-4R Mediate Allergic Reactions In Vitro

**Figure 7:**
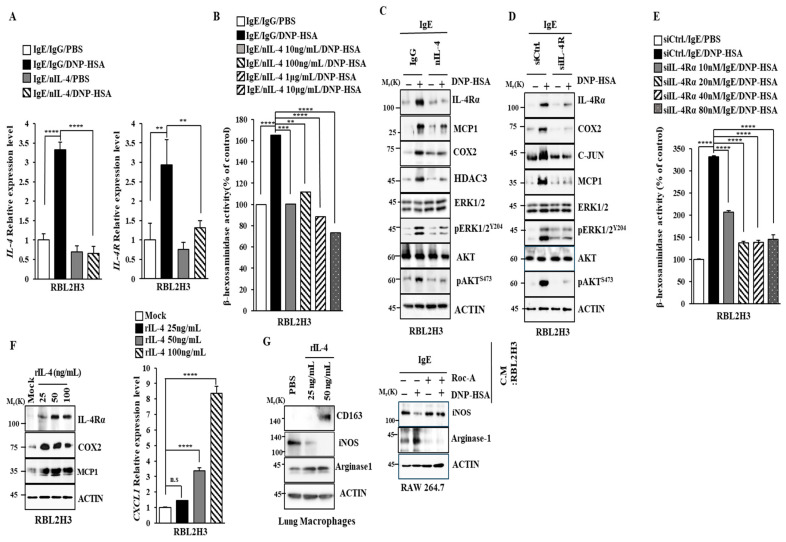
IL-4 mediates allergic reactions in vitro. (**A**) DNP-specific IgE (100 ng/mL)-sensitized RBL2H3 cells were pre-incubated with the indicated antibody (each at 0.2 μg/mL) for 2 h. nIL-4 denotes neutralizing IL-4 antibody. QRT-PCR was performed. **, *p* < 0.01; ****, *p* < 0.0001. (**B**) DNP-specific IgE (100 ng/mL)-sensitized RBL2H3 cells were pre-incubated with various concentrations of nIL-4 antibody or isotype-matched IgG antibody (0.2 μg/mL) for 2 h. β-hexosaminidase activity assays were performed. **, *p* < 0.01; ***, *p* < 0.001; ****, *p* < 0.0001. (**C**) DNP-specific IgE (100 ng/mL)-sensitized RBL2H3 cells were pre-incubated with nIL-4 antibody (0.2 μg/mL) or isotype-matched IgG antibody (0.2 μg/mL) for 2 h. (**D**) RBL2H3 cells were transfected with the indicated siRNA (each at 40 nM). The next day, cells were treated with DNP-specific IgE (100 ng/mL) for 16 h. Ctrl. denotes control siRNA. (**E**) RBL2H3 cells were transfected with the indicated siRNA. The next day, cells were treated with DNP-specific IgE (100 ng/mL) for 16 h. β-hexosaminidase activity assays were performed. ****, *p* < 0.0001. (**F**) RBL2H3 cells were treated with various concentrations of mouse recombinant IL-4 protein for 2 h, followed by immunoblot (**left**). QRT-PCR analysis was performed (**right**). N.S. denotes not significant. ****, *p* < 0.0001. (**G**) Lung macrophages isolated from BALB/C mice were treated with various concentrations of mouse recombinant IL-4 protein for 2 h, followed by immunoblot (**left**). DNP-specific IgE (100 ng/mL)-sensitized RBL2H3 cells were pre-incubated without or with Roc-A (1 μM) for 2 h. The culture medium was then added to RAW264.7cells for 24 h (**right**).

A blockade of IL-4R can alleviate symptoms of respiratory disease by decreasing the serum levels of IgG4/IgE [35]. A blockade of IL-4Rα can attenuate allergic skin inflammation induced by OVA by increasing IL-17A [36]. These reports imply the role of IL-4 in allergic reactions such as anaphylaxis. A blockade of IL-4 using neutralizing IL-4 antibody (nIL-4 Ab) decreased the transcriptional levels of IL-4 and IL-4R in antigen-stimulated RBL2H3 cells (Figure 7A). The nIL-4 antibody exerted negative effects on the increase in β-hexosaminidase activity (Figure 7B), hallmarks of allergic inflammation, and IL-4R (Figure 7C). The downregulation of IL-4R prevented the antigen from increasing the hallmarks of allergic inflammation (Figure 7D) and β-hexosaminidase activity (Figure 7E). Mouse recombinant IL-4 protein increased the expression levels of IL-4Rα, COX2, and MCP1 in RBL2H3 cells (Figure 7F). A cytokine array employing serum showed that PSA increased the expression level of CXCL1 in a mouse model. Mouse recombinant IL-4 protein increased CXCL1 mRNA expression in a dose-dependent manner (Figure 7F). The M2 polarization of macrophages contributes to the pathogenesis of anaphylaxis [37]. Lung macrophages displayed an increased expression of CD163, a marker of M2 macrophages, in response to mouse recombinant IL-4 protein (Figure 7G). However, mouse recombinant IL-4 protein decreased the expression of inducible nitric oxide synthase (iNOS), a marker of M1 macrophages (Figure 7G). A culture medium of antigen-stimulated RBL2H3 cells increased the expression of arginase1, a marker of M2 macrophages (Figure 7G). Roc-A prevented a culture medium of antigen-stimulated RBL2H3 cells from regulating the expressions of iNOS and arginase 1 (Figure 7G). Thus, IL-4 and IL-4R might mediate allergic reactions by promoting the M2 polarization of macrophages. These reports suggest that Roc-A can inhibit allergic inflammation in vitro by regulating the M2 polarization of macrophages.

### 2.7. IL-4 and IL-4R Mediate Anaphylaxis

**Figure 8:**
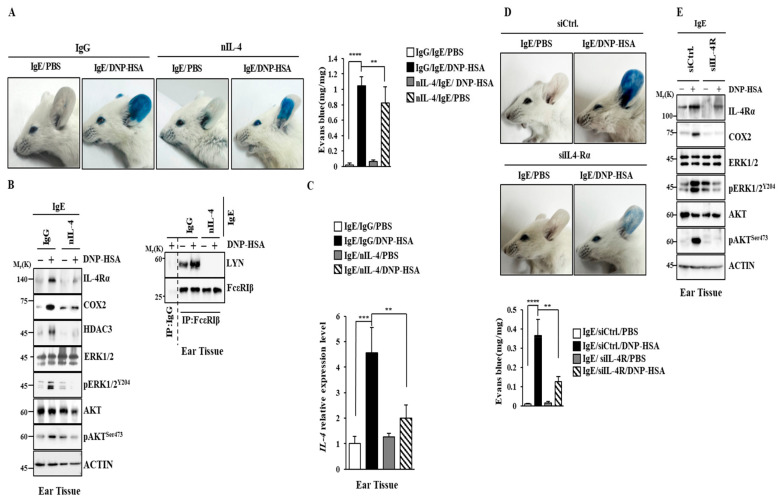
IL-4 and IL-4R are necessary for passive cutaneous anaphylaxis. (**A**) DNP-specific IgE (4 μg/kg) was intradermally injected while the indicated antibody (each at 50 μg/kg) was intravenously injected into BALB/C mice. The next day, intravenous injections of PBS or DNP-HSA (5 mg/kg) and 2% (*v*/*v*) Evans blue solution were performed. **, *p* < 0.01; ****, *p* < 0.0001. Each experimental group comprised four BALB/C mice. (**B**) Immunoblot and immunoprecipitation were performed. (**C**) QRT-PCR was performed. **, *p* < 0.01; ***, *p* < 0.001. (**D**) DNP-specific IgE (4 μg/kg) was intradermally injected while the indicated siRNA (each at 5 nmole/kg) was intravenously injected into BALB/C mice. The next day, intravenous injections of PBS or DNP-HSA (5 mg/kg) and 2% (*v*/*v*) Evans blue solution into BALB/C mice were performed. Each experimental group comprised four BALB/C mice. **, *p* < 0.01; ****, *p* < 0.0001. (**E**) Immunoblot was performed.

Next, the role of IL-4 in anaphylaxis was then examined. The results showed that nIL-4 prevented the antigen from increasing vascular permeability (Figure 8A). It also prevented the antigen from increasing the expression of IL-4R and hallmarks of allergic inflammation in a mouse model of PCA (Figure 8B). In addition, nIL-4 prevented the antigen from increasing IL-4 mRNA (Figure 8C). The downregulation of IL-4R also inhibited PCA (Figure 8D) and prevented the antigen from increasing hallmarks of allergic inflammation (Figure 8E). IL-4R was necessary for PSA (Figure S3A) and an increased expression of hallmarks of allergic inflammation (Figure S3C). PSA increased the transcriptional level of IL-4R (Figure S3B). Thus, IL-4 and IL-4R could mediate anaphylaxis.

### 2.8. miR-34a Inhibits Allergic Reactions In Vitro by Decreasing IL-4R Expression

**Figure 9:**
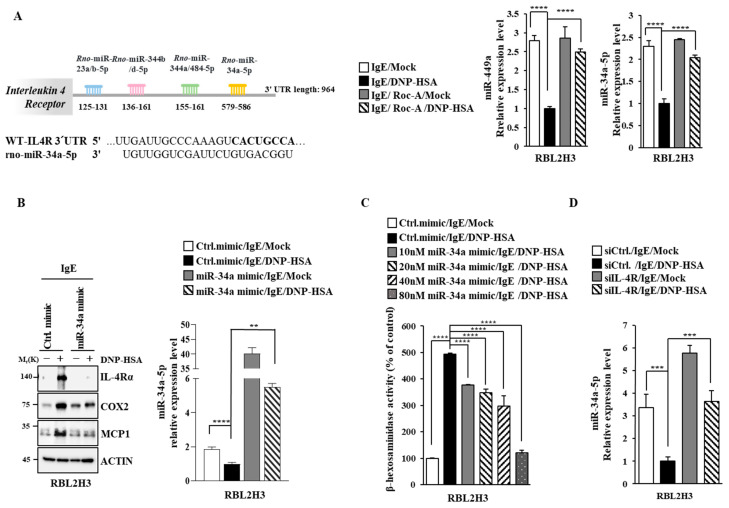
miR-34a-5p inhibits allergic reactions in vitro. (**A**) miRNAs that can bind to the 3′ UTR of IL-4R are shown. DNP-specific IgE (100 ng/mL)-sensitized RBL2H3 cells were treated without or with Roc-A (1 μM) for various time intervals. QRT-PCR was performed. ****, *p* < 0.0001. (**B**) RBL2H3 cells were transfected with the indicated mimic (each at 20 nM). The next day, cells were sensitized with DNP-specific IgE (100 ng/mL) for 24 h. Immunoblot and qRT-PCR were performed. **, *p* < 0.01; ****, *p* < 0.0001. (**C**) RBL2H3 cells were transfected with the indicated mimic. The next day, cells were sensitized with DNP-specific IgE (100 ng/mL) for 24 h, followed by stimulation with DNP-HSA (100 ng/mL) for 1 h. ****, *p* < 0.0001. (**D**) RBL2H3 cells were transfected with the indicated siRNA (each at 40 nM). The next day, cells were treated with DNP-specific IgE (100 ng/mL) for 24 h. QRT-PCR were performed. ***, *p* < 0.001.

Targetscan analysis predicted that miR-34a-5p and miR-344b-1-5p could regulate the expression of IL-4R by binding to the 3′ UTR of IL-4R (Figure 9A). Antigen stimulation decreased the expressions of miR-34a-5p and miR-449a, a family of miR-34a (Figure 9A). However, Roc-A restored the expressions of miR-34a-5p and miR-449a in antigen-stimulated RBL2H3 cells (Figure 9A). This suggests that miR-34a might exert an anti-allergic effect. In the present study, an miR-34a mimic prevented the antigen from increasing the expression levels of MCP1, COX2, and IL-4R (Figure 9B) and β-hexosaminidase activity in RBL2H3 cells (Figure 9C). The downregulation of IL-4R increased the expression of miR-34a-5p in antigen-stimulated RBL2H3 cells (Figure 9D). miR-34a and IL-4R could form a negative feedback loop to regulate allergic reactions in vitro.

### 2.9. IL-4R Docking Chemical Inhibits Allergic Reactions In Vitro

**Figure 10:**
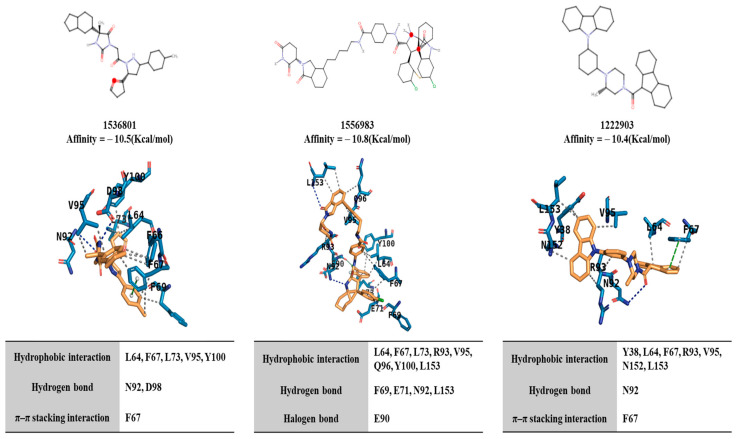
Potential binding of chemical compounds to IL-4R and analysis of their interactions. The upper panel presents the 2D structure of the compound, its name, and the calculated affinity by docking simulation. The middle panel shows the interactions between each compound and residues of IL-4R. These interaction analyses are described in the bottom panel.

We performed virtual screening to identify chemical compounds that could bind to IL4R. We hypothesized that these chemicals could disrupt the interaction between IL-4 and IL-4R. The top-ranked compounds were selected based on their docking results. Their interactions and poses were then analyzed (Figure 10). Compounds 1536801, 1556983, and 1222903 exhibited high docking affinity scores of −10.5, −10.8, and −10.4 Kcal/mole, respectively, indicating their potential to bind strongly to IL-4R. Interaction analysis revealed that these compounds formed various types of interactions with key residues in IL-4R. These compounds engaged in hydrophobic interactions with residues such as L64, F67, L73, R93, V95, Q96, Y100, and L153. Furthermore, 1536801 and 1556983 formed hydrogen bonds with residues such as F69, E71, N92, and L153, while 1222903 formed a hydrogen bond with N92. Interestingly, 1536801 and 1222903 also demonstrated π-π stacking interactions with F67, suggesting a robust and stable binding mode. The presence of these diverse interactions, including hydrophobic contacts, hydrogen bonds, and π-π stacking, highlights the potential of these compounds to effectively bind to IL-4R and potentially disrupt IL-4/IL-4R interaction.

**Figure 11:**
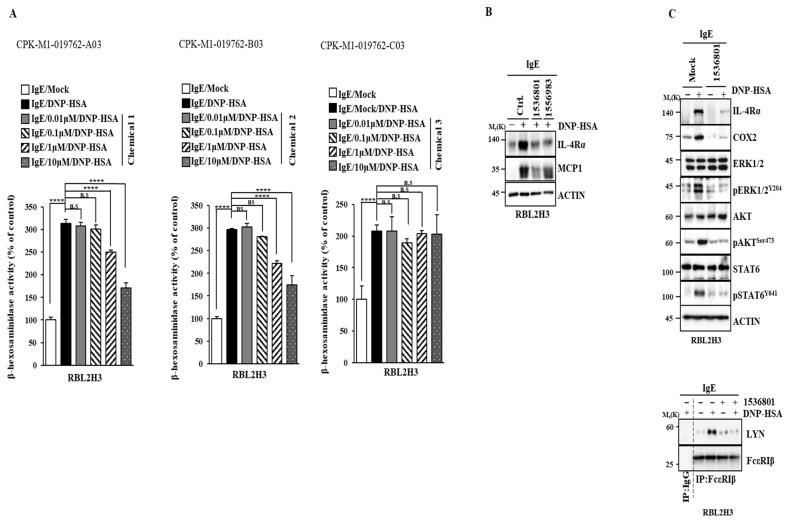
IL-4R docking chemical inhibits allergic reactions in vitro. (**A**) DNP-specific IgE (100 ng/mL)-sensitized RBL2H3 cells were treated without or with the indicated chemical for 2 h. β-hexosaminidase activity assays were performed. ****, *p* < 0.0001. N.S. denotes not significant. Chemical, 1536801; chemical 2, 1556983; chemical 3, 1222903. (**B**) DNP-specific IgE (100 ng/mL)-sensitized RBL2H3 cells were treated without or with the indicated chemical (each at 5 μM) for 2 h. Immunoblot was performed. (**C**) DNP-specific IgE (100 ng/mL)-sensitized RBL2H3 cells were treated without or with chemical 1536801 (5 μM) for 2 h.

Among the various chemicals tested, chemicals 1536801 and 1556983 prevented the antigen from increasing β-hexosminidase activity (Figure 11A) and the expressions of IL-4R and MCP1 in RBL2H3 cells (Figure 11B). Chemical 1536801 also prevented the antigen from increasing the phosphorylation levels of ERK, AKT, and STAT6 and prevented the antigen from inducing the binding of FcεRI to LYN in RBL2H3 cells (Figure 11C). Thus, IL-4R can serve as a target for developing anti-allergy drugs.

### 2.10. IL-4R Docking Chemical Inhibits Anaphylaxis

**Figure 12:**
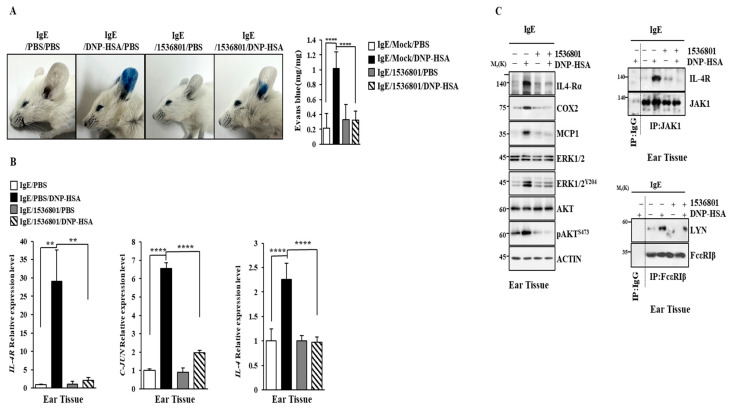
IL-4R docking chemical suppresses PCA. (**A**) DNP-specific IgE (4 μg/kg) was intradermally injected while chemical 1536801 (993.2 ng/kg) was intravenously injected into BALB/C mice. The next day, intravenous injections of PBS or DNP-HSA (5 mg/kg) and 2% (*v*/*v*) Evans blue solution were performed. ****, *p* < 0.0001. Each experimental group comprised four BALB/C mice. (**B**) QRT-PCR was performed. **, *p* < 0.01; **** *p* < 0.0001. (**C**) Immunoblot and immunoprecipitation were performed.

We next examined the effect of the IL-4R docking chemical on anaphylaxis. Chemical 1536801 prevented the antigen from enhancing vascular permeability in a mouse model of PCA (Figure 12A) and prevented the antigen from increasing the transcriptional levels of IL-4, IL-4R, and C-Jun (Figure 12B). Chemical 1536801 exerted negative effects on increases in hallmarks of allergic inflammation and inhibited the binding of FcεRI to LYN in a mouse model of PCA (Figure 12C). Chemical 1536801 also prevented the antigen from inducing the binding of IL-4R to JAK1 (Figure 12C).

**Figure 13:**
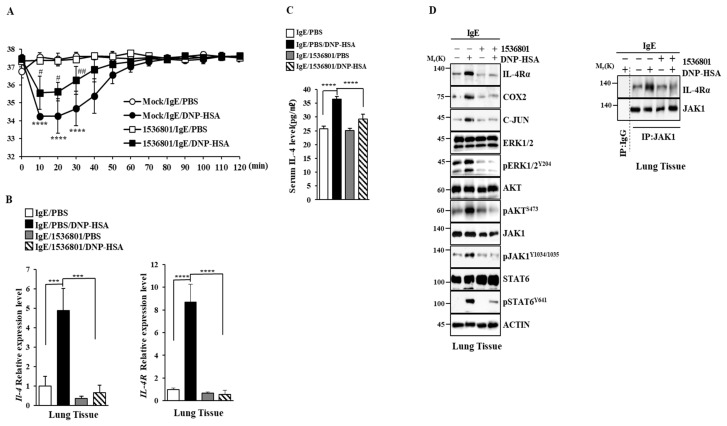
IL-4R docking chemical suppresses PSA. (**A**) Intravenous injections of DNP-specific IgE (4 μg/kg) and chemical 1536801 (993.2 ng/kg) were performed. The next day, intravenous injections of DNP-HSA (5 mg/kg) into BALB/C mice were performed and rectal temperatures were measured. Each experimental group comprised five mice. The means ± S.E. of three independent experiments are shown. ****, *p* < 0.0001, compared with IgE/PBS. # and, ##, compared with IgE/DNP-HSA. (**B**) Lung tissue lysates were subjected to qRT-PCR. ***, *p* < 0.001; ****, *p* < 0.0001. (**C**) Serum IL-4 ELISA was performed. ****, *p* < 0.0001. (**D**) Immunoblot and immunoprecipitation employing lung tissue lysates were performed.

Chemical 1536801 prevented the antigen from decreasing the rectal temperature in a mouse model of PSA (Figure 13A) and prevented the antigen from increasing the expressions of IL-4 and IL-4R (Figure 13B). Chemical 1536801 prevented the antigen from increasing the serum IL-4 level (Figure 13C) and prevented the antigen from increasing hallmarks of allergic inflammation (Figure 13D). These results confirm the validity of IL-4R as a target for developing anti-allergy drugs.

## 3. Discussion

Since Roc-A displayed anticancer activities, we first examined whether it could exert anti-allergic effects. Roc-A inhibited anaphylaxis both in vitro (Figure 1) and in vivo (Figure 2). In this study, we wanted to understand the mechanisms of allergic reactions in more detail. RNA sequencing analysis showed that IL-4 was one of the most highly upregulated genes by the antigen in RBL2H3 cells (Figure 3). RNA sequencing also showed that Roc-A decreased the expressions of IL-4 and IL-13 in antigen-stimulated RBL2H3 cells (Figure 3). IL-4 can mediate food allergy by inhibiting regulatory T cell function [38]. A blockade of IL-4/IL-13 signaling can suppress IgE production and Th2 cytokine responses in a mouse model of peanut allergy [39]. IL-4 blockade can suppress the secretion of Th2 cytokines in a mouse model of asthma [40]. These reports imply the potential role of IL-4 signaling in allergic reactions such as anaphylaxis. Roc-A decreased the transcriptional levels of IL-4 and IL-4R in antigen-stimulated RBL2H3 cells (Figure 4A). Therefore, Roc-A might inhibit allergic reactions by targeting IL-4 signaling.

RNA sequencing showed that antigen stimulation increased the expression of suppressor of cytokine signaling 3 (SOCS3) in RBL2H3 cells (Figure 3). Ovalbumin (OVA)-induced food allergy can increase the phosphorylation of STAT3 and activate SOCS3 [41]. Allergic airway inflammation is known to increase the expression of SOCS3 [42]. The STAT3/SOCS3 and mitogen activated-protein kinase (MAPK) pathways can mediate allergic asthma [43]. Atopic dermatitis (AD) is known to increase the expression levels of SOCS3 and IL-4 [44]. CXCL1 and SOCS3 play key roles in allergic airway inflammation [45]. It is probable that the activation of IL-4R signaling might lead to an increased expression of SOCS3. Thus, it is necessary to examine the role of SOCS3 in anaphylaxis in the future.

Atopic dermatitis (AD) can increase the level of CXCL1 by activating type 2 IL-4R [30]. CXCL1 is known to mediate OVA-induced food allergy [46]. We found that PSA increased the level of CXCL1 (personal observations). IL-4 signaling might increase CXCL1 expression to mediate allergic inflammations such as anaphylaxis. M2 macrophages enhance cervical cancer cell migration and invasion by secreting CXCL1 [47]. Mast cell migration into tumor lesions can enhance cancer progression [18]. CXCL1 might mediate cellular interactions involving mast cells, macrophages, and cancer cells. It is necessary to examine whether recombinant IL-4 protein can increase the level of CXCL1 in the future.

MAPK signaling can mediate anaphylactic reactions [9,10,48,49]. AKT, ERK1/2, and JNK are known to mediate PCA [50]. Roc-A prevented the antigen from increasing the phosphorylation of ERK and AKT in RBL2H3 cells (Figure 1D). ERK and AKT were necessary for increased β-hexosaminidase activity (Figure S1D,E) and the expression of IL-4R in antigen-stimulated RBL2H3 cells (Figure 5E). It will be necessary to identify downstream targets of ERK and AKT.

IL-4Rα mediates allergic asthma by inducing the M2 polarization of macrophages [51]. Protein kinase C (PKC)–IL-4–STAT6 signaling contributes to the pathogenesis of allergic airway inflammations [52]. M2-polarized macrophages can enhance the metastatic potential of osteosarcoma cells by activating Janus kinase (JAK2)/signal transducer and activator of transcription 3 (STAT3) signaling [53]. Recombinant IL-4 protein increased the expressions of CD163 and arghinase-1 but decreased the expression of iNOS in lung macrophages (Figure 7G). Antigen stimulation induced the binding of IL-4R to JAK1 (Figure 4C) and increased the phosphorylation of JAK1 (Figure 4C). It would be interesting to examine whether recombinant IL-4 protein could induce the binding of IL-4R to JAK1 and increase the phosphorylation of JAK1.

Type II IL-4R consists of IL-4Rα and IL-13Rα. RNA sequencing analysis showed that IL-13 was one of the genes most highly upregulated by antigen stimulation (Figure 3). Since IL-13 recruits members of the Janus Kinase family (JAK1, JAK2, and TYK2) to its receptor complex [54], JAK inhibitors might inhibit IL-4R signaling to suppress anaphylaxis. Since recombinant IL-4 protein increased the expression of markers of M2 macrophages (Figure 7F,G), recombinant IL-13 protein might also increase markers of M2 macrophages.

Allergic skin lesions involve the phosphorylation of JAK2 and STAT3 [55]. Gp130 can promote allergic conjunctivitis by increasing the phosphorylation levels of JAK2 and STAT3 [56]. The IL-4Rα–JAK2–STAT3 pathway can mediate OVA-induced allergic rhinitis [57,58]. JAK2/STAT6 signaling contributes to the pathogenesis of allergic airway inflammation [59,60]. We found that the phosphorylation of STAT6 was increased by antigen stimulation in RBL2H3 cells (Figure 11C). Since JAK2 contributes to the pathogenesis of allergic airway inflammation, it will be necessary to examine whether JAK2 inhibitors can suppress anaphylaxis.

Roc-A can inhibit autophagy by targeting Unc51-like autophagy activating kinase 1 (ULK1) and enhance NK-cell-mediated killing [61]. Allergic reactions are accompanied by an increase in autophagic flux [9,62]. It is probable that Roc-A can inhibit autophagic flux during allergic reactions such as anaphylaxis.

Sphingosine-1-phosphate (S1P) can increase the CXCL1 level via MAPK signaling in astrocytes [63]. Roc-A prevented the antigen from increasing the expression of CXCL1 in a mouse model of PSA (personal observations). Since PSA increased the CXCL1 level, it is reasonable that S1P signaling might mediate allergic reactions. The S1PR2–MCP1 axis could mediate neuro inflammation in a mouse model of encephalopathy [64]. S1PR2 can mediate cerebrovascular inflammation by regulating the expression of CXCL1 [65]. S1PR2 is known to contribute to the pathogenesis of allergic asthma by inhibiting autophagy [66]. S1PR2 signaling can induce airway T cell infiltration in mouse models of acute allergic reactions [67]. S1PR2 signaling can mediate mast-cell-promoted inflammation by increasing the expression of VEGF-A [68]. These reports imply a role of S1PR2 in allergic inflammations such as anaphylaxis. IL-4 can induce the phosphorylation of JAK2 and STAT6 in an S1PR2-dependent manner in macrophages [69]. S1P can induce M2 polarization via IL-4 secretion to exert an anti-atherogenic effect [70]. These reports suggest that cross talk between S1PR2 and IL-4R may mediate allergic inflammations such as anaphylaxis.

Previous reports have indicated roles of miRNAs in allergic inflammation [9,59]. In the present study, an miR-34a mimic suppressed allergic reactions in vitro. We found that miR-34a and IL-4R formed a negative feedback loop to regulate allergic reactions. It is necessary to examine whether miR-34a can directly bind to the 3ʹ UTR of IL-4R in the future. miR-34a can directly regulate the expression of MYCN and suppress anaphylaxis [71]. It will be also necessary to identify downstream targets of the miR-34a mimic. These genes will be helpful for better understanding IL-4-promoted anaphylaxis. It is reasonable that IL-4R signaling might increase MYCN expression during allergic inflammation.

In this study, we identified IL-4R docking chemicals. Among the identified chemicals, chemical 1536801 suppressed anaphylaxis (Figure 12 and Figure 13). Downstream targets of chemical 1536801 might serve as targets for developing anti-allergy therapeutics. The mechanistic details of anti-allergic effects by chemical 1,535,801 will give valuable insights into the mechanisms associated with allergic reactions such as anaphylaxis. Identifying proteins that can bind to chemical 1536801 is needed in the future.

In this study, we did not find cytotoxic effects of Roc-A. This implies that Roc-A can be used in combination with current allergy therapeutics. In this study, we found that miR-34a negatively regulated in vitro allergic reactions by decreasing the expression of IL-4R. It is interesting to examine whether Roc-A can enhance the effect of miR-34a. It is probable that Roc-A can enhance the inhibitory effect of nIL-4 antibody on allergic reactions. Since Roc-A can bind to IL-4R, it will be necessary to further identify molecules that can bind to Roc-A. These molecules can be employed for combination therapy involving Roc-A. It is probable that Roc-A may induce unwanted side effects. It will be necessary to modify Roc-A in a way to reduce the side effects associated with it. It will be also necessary to develop a novel delivery system for Roc-A.

## 4. Materials and Methods

### 4.1. Materials

Chemicals were purchased from Sigma-Aldrich (St. Louis, MO, USA). SiRNAs and primers were purchased from Bioneer Company (Daejeon, Republic of Korea). Mouse control mimic and miR-34a-5p mimic were obtained from Dharmacon Inc. (Lafayett, CO, USA). HRP-conjugated goat anti-rabbit IgG was purchased from ENZO Life science (ADI-SAB-300-J, New York, NY, USA). HRP-conjugated anti-mouse IgG was purchased from Cell Signaling (7076, Danvers, MA, USA). Recombinant mouse IL-4 protein was purchased from R&D system (404-ML-010/CF, Minneapolis, MN, USA). Neutralizing IL-4 antibody (nIL-4) was purchased from Invitrogen (AMC0044, Waltham, MA, USA). For in vivo injections, nIL-4 was purchased from BioXCell (BE0045, Lebanon, NH, USA).

### 4.2. Cell Culture

RBL2H3 cells were purchased from ATCC (Manassas, VA, USA). We purchased RAW264.7 cells from Korea Cell Line Bank (Seoul, Republic of Korea). Cells were grown in Dulbecco’s modified Eagle’s medium (DMEM) supplemented with 10% fetal bovine serum (FBS), 2 mM L-glutamine, and 1X penicillin/streptomycin. Cultures were maintained in 5% CO_2_ at 37 °C. Lung macrophages and bone marrow mast cells (BMMCs) were isolated based on standard procedures [9]. BMMCs were isolated from the femoral and tibia bone marrow cells of BALB/C mice. BMMCs were grown in DMEM supplemented with 10% FBS and IL-3 (30 ng/mL). Cell number and viability were determined using trypan blue staining. The cells were tested using an e-Myco^TM^ plus Mycoplasma PCR Detection Kit (iNtRON, Seongnam, Republic of Korea, cat # 25237) to ensure that they were mycoplasma free. Sensitization with DNP-specific IgE was performed when cells reached a confluence of 80%. When cells reached a confluence of 90%, they were treated with antigen. DNP-specific IgE (100 ng/ml) was added to DMEM containing 10% fetal bovine serum. The antigen (DNP-HSA) was added to a culture medium without FBS. DNP-specific IgE and DNP-HSA were dissolved in PBS. The AKT inhibitor, ERK inhibitor, and Roc-A were dissolved in DMSO.

### 4.3. Animals

Female BALB/C mice (~20 g, 8 weeks) were purchased from Nara Biotech (Seoul, Republic of Korea). The mice were co-housed under specific pathogen-free conditions (20–26 °C, 150–300 lux, 40–60% humidity) with a 14–10 h light–dark period. The animals were allowed free access to food and water. All animal experiments were approved by the Institutional Animal Care and Use Committee (IACUC) of the Kangwon National University and followed the ARRIVE guidelines. Animal euthanasia was performed using CO_2_ gas at a 30–70% displacement rate of the cage volume/min using a flow meter according to the American Veterinary Medical Association (AVMA) euthanasia guideline 43.

The mice had a minimum of 7 days of acclimation before being used for the experiments. Newly received mice were housed in cages that were separate from the existing animals. Mice were maintained as specific-pathogen-free (SPF). We generally followed protocols of health evaluations for the experimental animals.

### 4.4. Reactive Oxygen Species Measurement

DCFH-DA solution (10 μM) was added to the RBL2H3 cells twenty minutes after the addition of DNP-HSA. The fluorescence level of the 2′, 7′-dichlorofluorescein (DCF) was quantified by a fluorescence microscope.

### 4.5. RNA Sequencing and Analysis

TRIzol^®^ RNA Isolation Reagents (FAVORGEN BIOTECH, Chung-Chem 1st Rd. Kaohsiung, Taiwan) were used for the extraction of the total RNA. An Illumina TruSeq Stranded mRNA Sample Preparation kit (Illumina, San Diego, CA, USA) was used for constructing a messenger RNA sequencing library. All libraries were quantified by qPCR (CFX96, Bio-Rad, Hercules, CA, USA) and sequenced on NextSeq500 sequencers (Illumina) with a paired-end 75 bp plus single 8 bp index read run. To quantify the mapped reads on the reference genome into gene expression values, Cuffquant in Cufflinks with the strand-specific library option and other default options was used. The analysis of the differentially expressed genes was performed using the Cuffdiff software (Version 0.8.0) with the strand-specific library option [72]. For a comparison of the differential expression files, clustering of the normalized expression values of the differentially expressed genes was performed by in-house R scripts. A heatmap of expression values of the selected DEGs in log10 (FPKM) units was compared across genes and samples (fold changes > 2 and *p*-value < 0.05). GO and KEGG enrichment analyses were performed by g:Profiler2 ver. 0.2. The RNA seq data sets can be found at the NCBI’s Sequence Read Archive (https://www.ncbi.nlm.nih.gov/sra (accessed on 23 August 2023)) (PRJNA1010564).

### 4.6. Transfections

Small interfering RNAs (SiRNAs) and microRNA (miR) mimics were purchased from Bioneer Company (Daejeon, Republic of Korea). For transfections, JetPEI^®^ (Polyplus, New York, NY, USA, cat.201-10G) was used. RBL2H3 cells were transfected with siRNA (each at 10 nM) or miR mimic (each at 10 nM) for 24 h. At 24 h after seeding, the cells were transfected with JetPEI^®^ (Polyplus, cat.201-10G). All transfections were performed in the presence of serum. The sequences of mimics and siRNAs are shown in Supplementary Tables S1 and S2, respectively. Transfections were performed when the cells reached a confluence of 50–60%.

### 4.7. β-Hexosaminidase Activity Assays

β-hexosaminidase activity assays were performed as described [9].

### 4.8. Quantitative Real-Time PCR

TRIzol reagent (Thermo Fisher, Waltham, MA, USA) was used for isolating total RNAs. Then, 1 μg total RNA was subjected to reverse transcription employing a pre-mix reverse transcription kit (iNtRon Biotechnology, Kyunggi, Republic of Korea). A quantitative real-time polymerase chain reaction (RT-PCR) was performed using the synthesized cDNA and a SYBR-green mixture containing the Rox dye (Excel Taq™ 2X Fast Q-PCR Master Mix) (SMOBIO, Hsinchu, Taiwan) in a StepOne^TM^ Real-Time PCR System (Thermo Fisher, Waltham, MA, USA). The PCR conditions were 40 cycles of denaturation for 3 s at 95 °C, annealing for 30 s at 60 °C, and extension for 30 s at 60 °C. The primer sequences used are listed in Supplementary Table S3. The relative mRNA levels were determined using the ΔΔ-Ct value and normalized to that of mouse or rat actin mRNA.

### 4.9. Chromatin Immunoprecipitation Assays

Cells were cross-linked in 4% formaldehyde solution for 10 min, and DNA was isolated. Lysates were extracted and chromatin was sheared by sonication to 400–500 bp, followed by centrifugation to remove cell debris. C-Jun antibody (2 μg/mL) and isotype-matched IgG control antibody or actin antibody (2 μg/mL) were used for the ChIP assay. Incubation with antibodies continued for 16 h at 4 °C. Immunoprecipitation was performed with protein A/G PLUS-Agarose (Santacruz, Dallas, TX, USA, sc-2003) at 4 °C for 14 h. PCR was conducted with specific primers of the IL-4 promoter-1 (5′-GAGGACAAGCTGAGCAACAG-3′ (sense) and 5′-ACCAATTAATTCCCCAGCGG-3′ (antisense)), IL-4 promoter-2 (5′-CCGCTGGGGAATTAATTGGT-3′ (sense) and 5′-GGGCAAGGTTGACGATTGTT-3′ (antisense)), and IL-4 promoter-3 (5′-CCTCATTTCATGGTCCTGCC-3′ (sense) and 5′-CTTATCAGCGTAGGGTTGCC-3′ (antisense)) sequences. IL-4R promoter-1 (5′- CTGCCACTGAGCTGCATTC-3′ (sense) and 5′-TGCACTGTGACACCATGAGA-3′ (antisense)), IL-4R promoter-2 (5′-TGGTTATGATGGACCGGTGT-3′ (sense) and 5′-AAGGTTGTCACCCTGCCATA-3′ (antisense)), and IL-4R promoter-3 (5′-TAGAGACCCAGACAGAGGGG-3′ (sense) and 5′-AAATGACGGTAGACCCCAGG-3′ (antisense)) were used to determine the binding of C-Jun.

### 4.10. ELISA

The level of serum IL-4 was determined by EKISA kit (Abcam, Cambridge, UK, ab100710).

### 4.11. Immunoblot and Immunoprecipitation

Cells were lysed in lysis buffer (50 mM Tris-HCl (pH, 6.8), 150 mM NaCl, 1% NP-40, 50 mM dithiothreitol) supplemented with 1 mM sodium ortho vanadate and 1% protease inhibitor cocktail (Roche, Basel, Switzerland). Lysates were cleared by centrifugation (15,000× *g*, 4 °C, 15 min), and protein concentrations were quantified with the Bicinchoninic Acid (BCA) Protein Assay Kit (GenDEPOT, Katy, TX, USA). Cell lysates, separated by sodium dodecyl sulfate-polyacrylamide gel electrophoresis, were transferred onto polyvinylidene fluoride (PVDF) membranes. Following blocking in TBS-T with 2% BSA for 1hr, incubation with the indicated primary antibodies continued overnight at 4 °C. After washing three times in TBS-T, the membranes were incubated with the respective secondary antibodies for 2 h. After washing with TBS-T, immunodetection was performed using West-Q Pico ECL Solution (GenDEPOT, Katy, TX, USA). Chemiluminescence images were obtained using an Amersham™ ImageQuant™ 500 imaging system (Cytiva, Washington, DC, USA).

The following primary antibodies were used: HDAC3 (#3949, Cell Signaling), COX2 (#12282, Cell Signaling), MCP1 (ab25124, Abcam), ERK1/2 (#4695, Cell Signaling), pERK^T204^ (#4370, Cell Signaling), AKT (#4691, Cell Signaling), pAKT^Ser473^ (#4060, Cell Signaling), FcεRIβ (sc-393789, Santa Cruz, Dallas, TX, USA), LYN ((#2732, Cell Signaling), CD163 (ab182422, Abcam), iNOS (ab115819, Abcam), IL-4 receptor α (#bs-23579R, Bioss, Woburn, MA, USA), JAK1 (#50996S, Cell Signaling), pJAK1^Y1034/1035^ (#3331S, Cell Signaling), eIF4A (#2013S, Cell Signaling), C-Jun (#9165S, Cell Signaling), YY1 (#sc-7341, Santa Cruz), Arginase1 (#93668, Cell Signaling), and ACTIN (A2228, Sigma). For the isolation of tissue lysates, tissue was frozen in liquid nitrogen and homogenized with RIPA buffer. Vortexing and centrifugation at 10,000× *g* for 15 min at 4 °C followed this. The supernatant was used as a tissue lysate for immunoblot and immunoprecipitation.

### 4.12. Passive Cutaneous Anaphylaxis

DNP-specific IgE (4 μg/kg) was injected intradermally into the ear while Roc-A (993.2 ng/kg) was intravenously injected. The next day, an intravenous injection of PBS or DNP-HSA (5 mg/kg) along with 2% (*v*/*v*) Evans blue solution was performed on the BALB/C mice. To examine the effect of IL-4, DNP-specific IgE (4 μg/kg) was injected intradermally into the ear while isotype-matched IgG (20 μg/kg) or nIL-4 antibody (50 μg/kg) was intravenously injected. To determine the effect of IL-4R, DNP-specific IgE (4 μg/kg) was injected intradermally into the ear while negative-control siRNA (5 nmole/kg) or IL-4R siRNA (5 nmole/kg) were intravenously injected. To determine the effect of chemical 1536801, the BALB/C mice were intradermally injected with DNP-IgE (4 μg/kg) along with an intravenous injection with chemical 1536801 (993.2 ng/kg).

### 4.13. Passive Systemic Anaphylaxis

DNP-specific IgE (4 μg/kg) and Roc-A (505.567 μg/kg) were injected intravenously. Twenty-four hours later, DNP-HSA (5 mg/kg) was intravenously injected into BALB/C mice. Rectal temperatures were measured using a digital thermometer. To determine the effect of IL-4R on PSA, the BALB/C mice were intravenously injected with DNP-specific IgE (4 μg/kg) along with control siRNA or IL-4R siRNA (5 nmole/kg). Twenty-four hours later, the BALB/C mice were given an intravenous injection with PBS or DNP-HSA (5 mg/kg).

### 4.14. In Silico Molecular Docking Simulation

To verify the binding of Roc-A to IL-4R, an AlphaFold model of mouse IL-4R was used for a docking simulation [73]. The docking simulation was performed using AUTODOCK VINA [74], and the search area was set by the interaction site between IL-4R and IL-4 previously described in [75]. As ligands, two peptide molecules (ITLQEIIKT and RFLKRLDRN), which were extracted from IL-4 (PDB ID: 1IAR), were used. The best docking pose of Roc-A was analyzed by the Protein–Ligand Interaction Profiler [76]. The visualization of protein structures and their interactions was analyzed by PyMOL (The PyMOL Molecular Graphics System, Version 2.0 Schrödinger, LLC., New York, NY, USA). In the case of virtual screening, a compound library was obtained from the Korea Chemical Bank. The best docking poses of Roc-A and the other potential hit compounds selected by affinity rank were analyzed using the Protein–Ligand Interaction Profiler [76]. The visualization of protein structures and their interactions was analyzed by PyMOL (The PyMOL Molecular Graphics System, Version 2.0 Schrödinger, LLC.).

### 4.15. Statistical Analysis

Data were analyzed and graphed using the GraphPad Prism statistics program (GraphPad Prism Software version 7). The results are presented as means ± S.E. One-way ANOVA was carried out for comparisons among multiple groups. *p*-values of < 0.05 were considered to indicate a statistical difference.

## 5. Conclusions

Our results provide evidence that IL-4R signaling can serve as a target for developing anti-allergy drugs. It will be interesting to identify downstream targets of IL-4R. miRNAs that can bind to the 3′ UTR of IL-4R might be developed as anti-allergy drugs. Our results suggest that cross talk between IL-4R and S1PR2 might mediate allergic reactions. It is probable that Roc-A can bind to S1PR2. The identification of more chemicals that can bind to IL-4R and/or S1PR2 might give clues for developing anti-allergy therapeutics.

## Data Availability

Data are contained within the article and Supplementary Materials. All data are available upon request to the corresponding author. All sequence data are deposited at the NCBI Sequence Read Archive (SRA) (https://www.ncbi.nlm.nih.gov/sra (accessed on 23 August 2023)) (PRJNA1010564).

## Supplementary Materials

The following supporting information can be downloaded at: https://www.mdpi.com/article/10.3390/molecules30040840/s1, Figure S1: ROS, ERK, and Akt mediate allergic reactions in vitro. (A) DNP-specific IgE (100 ng/mL)-sensitized RBL2H3 cells were treated without or with NAC (500 μM) for 2h. cells were subjected to β-hexosaminidase activity assays. *, *p* < 0.05; ****, *p* < 0.0001. (B) DNP-specific IgE (100 ng/mL)-sensitized RBL2H3 cells were treated without or with AKT inhibitor VIII (500 μM) for 2h. DCFH-DA (10 μM) was added 20 minutes after the addition of DNP-HSA (100 ng/mL). *, *p* < 0.05; ****, *p* < 0.0001. (C) Same as (B) except that immunoblot was performed. (D) The β-hexosaminidase activity assays were performed. **, *p* < 0.01; ***, *p* < 0.001. (E) DNP-specific IgE (100 ng/mL)-sensitized RBL2H3 cells were treated without or with PD98059 (20 μM) for 2h. ***, *p* < 0.001; ****, *p* < 0.0001; Figure S2: C-Jun directly regulates the expression of IL-4. (A) Potential binding sites for transcription factors in the promoter sequences of IL-4R are shown (upper). DNP-specific IgE (100 ng/mL)-sensitized RBL2H3 cells were treated without or with roc-A (1 μM) for 2 h, followed by stimulation with DNP-HSA (100 ng/mL). ChIP assays were performed (lower). (B) At 24 h after transfection with the indicated siRNA, cells were sensitized with DNP-specific IgE (100 ng/mL) for another 24 h, followed by stimulation with DNP-HSA (100 ng/ml) for 1 h. The β-hexosaminidase activity assays were performed. **, *p* < 0.01; ****, *p* < 0.0001. N.S. denotes not significant. (C) At 24 h after transfection with the indicated siRNA (40nM), cells were sensitized with DNP-specific IgE for another 24 h, followed by stimulation with DNP-HSA (100 ng/mL) for 1 h. QRT-PCR assays were performed. **, *p* < 0.01; ***, *p* < 0.001; ****, *p* < 0.0001. (D) Same as (C) except that immunoblot was performed. Figure S3: IL-4R is necessary for passive systemic anaphylaxis. (A) Intravenous injection of the indicated siRNA (each at 3 μg/kg) into BALB/C mice was performed. The next day, BALB/C mice were given an intravenous injection of DNP-specific IgE (0.5 μg/kg). The following day, an intravenous injection of DNP-HSA (250 μg/kg) into BALB/C mice was performed and rectal temperatures were measured. Each experimental group is comprised of five mice. The means ± S.E. of three independent experiments were shown. ***, *p* < 0.001, compared with IgE/siCtrl./DNP-HSA; ####, *p* < 0.0001, compared with IgE/siCtrl. N.S. denotes not significant. (B) Lung tissue lysates were subjected to qRT-PCR. ****, *p* < 0.0001. (C) Immunoblot and immunoprecipitation were performed. Table S1: The sequences of microRNA mimics; Table S2: The sequences of SiRNAs; Table S3: Primer sequences for qRT-PCR.
