# Supplementary material for: Rocaglamide Suppresses Allergic Reactions by Regulating IL-4 Receptor Signaling
Source: Molecules. 2025 Feb 11;30(4):840. doi: 10.3390/molecules30040840 (PMC11858170; doi:10.3390/molecules30040840)
Supplement: Supplementary file 1 [file molecules-30-00840-s001.zip › Supplementary figures.pdf]

Figure S1

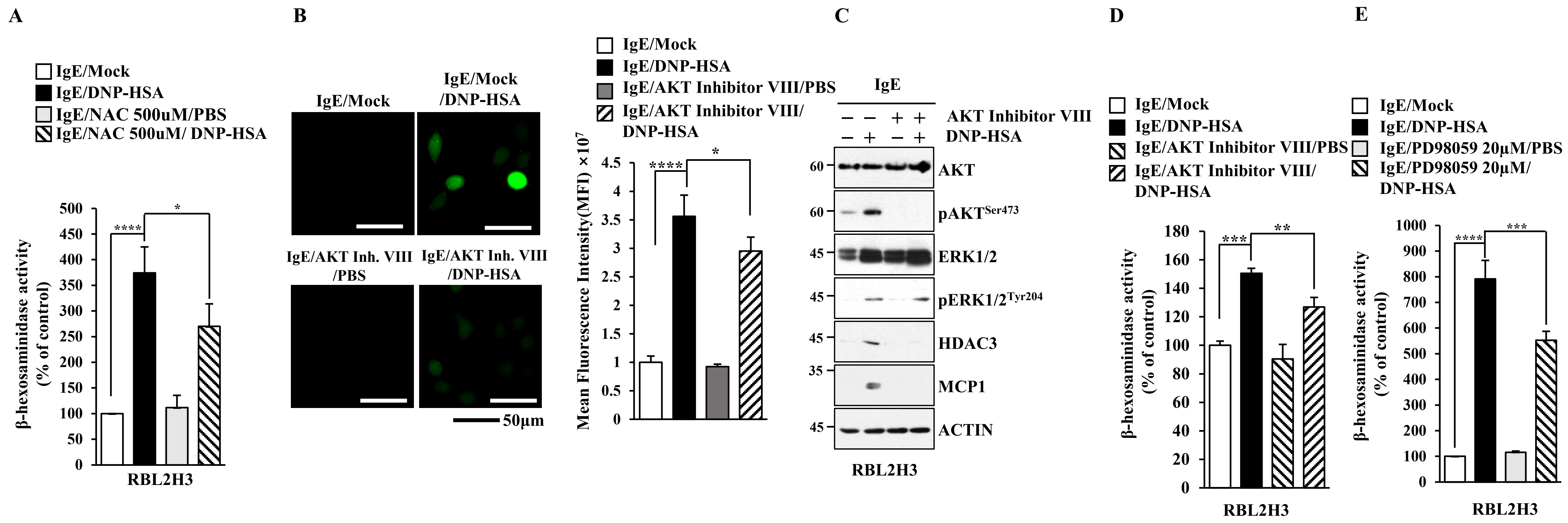

A
IgE/Mock
IgE/DNP-HSA
IgE/NAC 500uM/PBS
IgE/NAC 500uM/ DNP-HSA
β-hexosaminidase activity (% of control)
RBL2H3
B
IgE/Mock
IgE/Mock /DNP-HSA
IgE/AKT Inh. VIII /PBS
IgE/AKT Inh. VIII /DNP-HSA
50μm
IgE/AKT Inhibitor VIII/PBS
IgE/AKT Inhibitor VIII/ DNP-HSA
Mean Fluorescence Intensity(MFI) ×10^7
C
IgE
AKT Inhibitor VIII
DNP-HSA
AKT
pAKT Ser473
ERK1/2
pERK1/2 Tyr204
HDAC3
MCP1
ACTIN
RBL2H3
D
E
IgE/PD98059 20μM/PBS
IgE/PD98059 20μM/ DNP-HSA

Figure S2

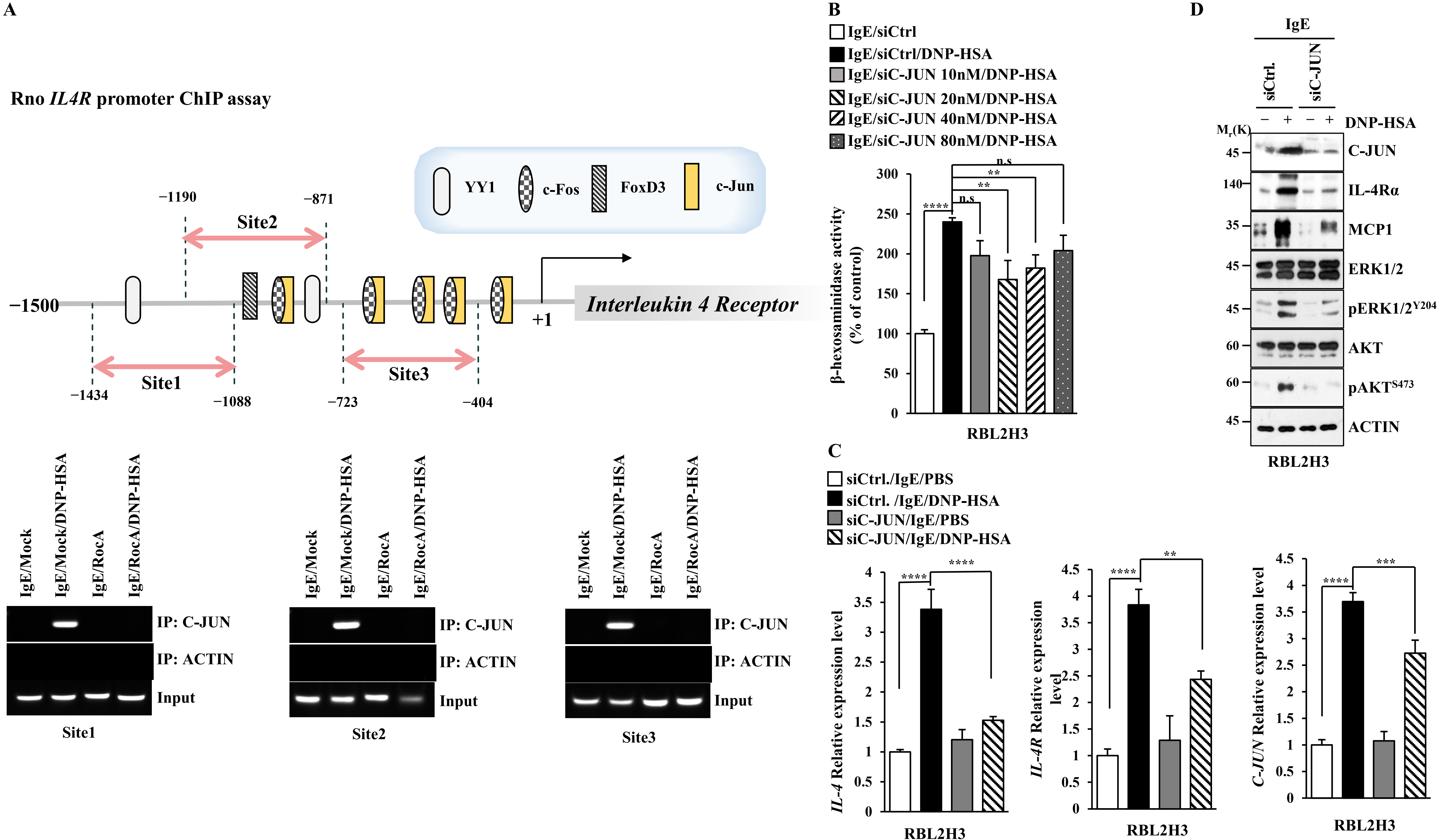
A
Rno IL4R promoter ChIP assay
YY1
c-Fos
FoxD3
c-Jun
−1190
−871
Site2
−1500
Interleukin 4 Receptor
+1
Site1
Site3
−1434
−1088
−723
−404
IgE/Mock
IgE/Mock/DNP-HSA
IgE/RocA
IgE/RocA/DNP-HSA
IP: C-JUN
IP: ACTIN
Input
Site1
Site2
Site3
B
IgE/siCtrl
IgE/siCtrl/DNP-HSA
IgE/siC-JUN 10nM/DNP-HSA
IgE/siC-JUN 20nM/DNP-HSA
IgE/siC-JUN 40nM/DNP-HSA
IgE/siC-JUN 80nM/DNP-HSA
β-hexosaminidase activity (% of control)
RBL2H3
C
siCtrl./IgE/PBS
siCtrl. /IgE/DNP-HSA
siC-JUN/IgE/PBS
siC-JUN/IgE/DNP-HSA
IL-4 Relative expression level
IL-4R Relative expression level
C-JUN Relative expression level
RBL2H3
D
IgE
siCtrl.
siC-JUN
DNP-HSA
Mr(K)
C-JUN
IL-4Rα
MCP1
ERK1/2
pERK1/2Y204
AKT
pAKTS473
ACTIN
RBL2H3

Figure S3

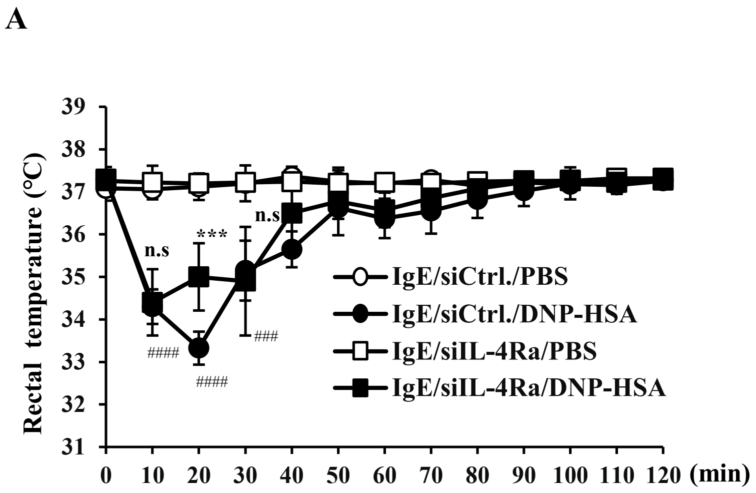
A
Rectal temperature (°C)
39
38
37
36
35
34
33
32
31
0
10
20
30
40
50
60
70
80
90
100
110
120
(min)
n.s
***
n.s
####
####
###
IgE/siCtrl./PBS
IgE/siCtrl./DNP-HSA
IgE/siIL-4Ra/PBS
IgE/siIL-4Ra/DNP-HSA

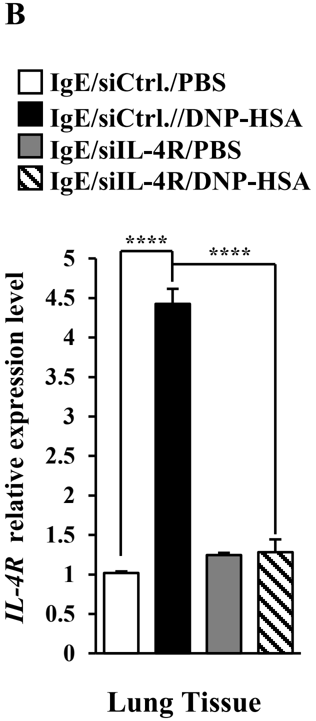
B
IgE/siCtrl./PBS
IgE/siCtrl.//DNP-HSA
IgE/siIL-4R/PBS
IgE/siIL-4R/DNP-HSA
IL-4R relative expression level
****
****
5
4.5
4
3.5
3
2.5
2
1.5
1
0.5
0
Lung Tissue

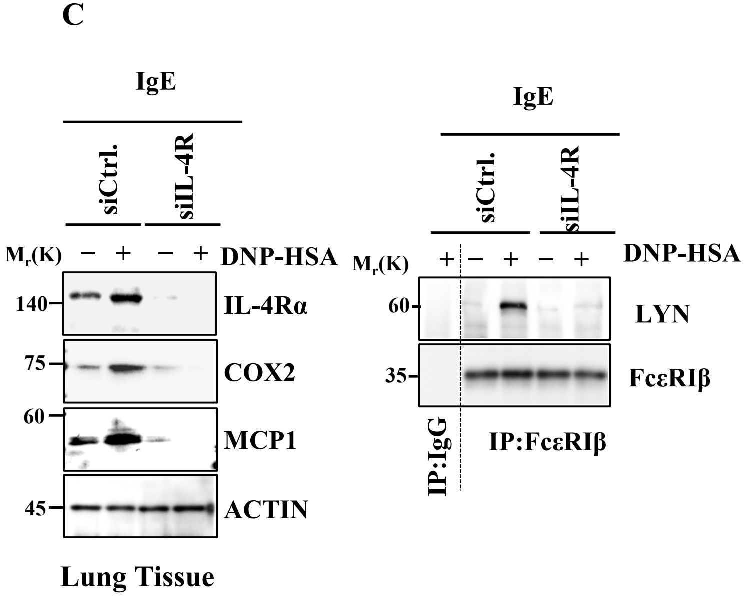
C
IgE
siCtrl.
siIL-4R
Mr(K)
DNP-HSA
140
IL-4Rα
75
COX2
60
MCP1
45
ACTIN
Lung Tissue
IgE
siCtrl.
siIL-4R
Mr(K)
DNP-HSA
60
LYN
35
FcεRIβ
IP:IgG
IP:FcεRIβ
