# Supplementary material for: Rocaglamide Suppresses Allergic Reactions by Regulating IL-4 Receptor Signaling
Source: Molecules. 2025 Feb 11;30(4):840. doi: 10.3390/molecules30040840 (PMC11858170; doi:10.3390/molecules30040840)
Supplement: Supplementary file 1 [file molecules-30-00840-s001.zip › Supplementary tables.pdf]

Supplementary Table 1. The sequences of microRNA mimics

Name	Company	Sequence (5'-3')
Negative control	Bioneer	AccuTarget™ miRNA Negative Control, mimic #1 DNA Oligo
miR-34a-5p	Bioneer	UGGCAGUGUCUUAGCUGGUUGU

Supplementary Table 2. The sequences of SiRNAs

Name	Company	Sequence (5'-3')
Negative control	Bioneer	AccuTarget™ Negative Control siRNA (Cat. SN-1002)
SiIL4R (Rat, Mouse)	Bioneer	Sense: GAGAACGUUCAUGUCAGUG<br>Antisense: CACUGACAUGAACGUUCUC
SiJUN (Rat)	Bioneer	Sense: GAGAAGAGGAACCUAUACU<br>Antisense: AGUAUAGGUUCCUCUUCUC

Supplementary Table 3. Primer sequences for qRT-PCR

Name	Sequence (5'-3')
U6	TGGCCCCTGCGCAAGGATG
miR-34a-5p	TGGCAGTGTCTTAGCTGGTTGT
miR-449a	TGGCAGTGTATTGTTAGCTGGT
IL-4 (Mouse)	F: GGAGATGGATGTGCCAAACG<br>R: GAAGCACCTTGGAAGCCCTA
IL-4 (Rat)	F: CGGTATCCACGGATGTAACG<br>R: GGTGCATGGAGTCCCTTTTT
IL-4R (Mouse)	F: ACGTGGTACAACCACTTCCA<br>R: GAACAGGCAAAACAACGGGA
IL-4R (Rat)	F: TTCCTAACCAGTCCCCCAAC<br>R: GCATTCTGGGGTGCTATTGG
c-Jun (Rat)	F: TCAGTAGCTGGCGCTGG<br>R: GCCAACCTCAGCAACTTCAA
CXCL1 (Rat)	F: GGGATTCACTTCAAGAACATCCAG<br>R: CTATGACTTCGGTTTGGGTGC
Actin (Mouse)	F: ATGTGGATCAGCAAGCAGGA<br>R: CTAGAAGCACTTGCGGTGC

Actin (Rat)	F: ACCCGCGAGTACAACCTTCT<br>R: TCGTCATCCATGGCGAACT
