# Supplementary material for: Rocaglamide Suppresses Allergic Reactions by Regulating IL-4 Receptor Signaling
Source: Molecules. 2025 Feb 11;30(4):840. doi: 10.3390/molecules30040840 (PMC11858170; doi:10.3390/molecules30040840)

qRT-PCR

Fig.4A RBL2H3_Rocaglamide A

Sample / Target gene	IgE/Mock/PBS	IgE/Mock/DNP-HSA	IgE/Roc-A/PBS	IgE/Roc-A/DNP-HSA
**IL-4**	1.058920146	3.275931165	1.002898835	2.229846123
	0.970539927	3.156395589	1.138512354	2.244427329
	0.970539927	3.328661115	1.076921814	2.324531228
**IL-4R**	2.006938853	15.92319142	0.139350874	2.120111567
	0.835121671	24.94859505	0.114710885	3.300817797
	0.157939476	20.29373668	0.182822276	3.474151782

Fig.4A RBL2H3_AKT Inh.VIII

Sample / Target gene	IgE/Mock/PBS	IgE/Mock/DNP-HSA	IgE/AKT Inh.VIII/PBS	IgE/AKT Inh.VIII/DNP-HSA
**IL-4**	1.156153533	3.247216214	1.191113822	1.230907412
	0.94467759	3.175903019	1.47103824	1.269589265
	0.899168877	3.231309169	1.198538142	1.308924183
**IL-4R**	1.167170141	3.432701974	0.849593683	0.979493934
	1.005339551	3.631987918	1.012157089	2.015400398
	0.827490308	4.375436671	1.383061242	2.247022509

Fig.4F RBL2H3_PD98059

Sample / Target gene	IgE/Mock/PBS	IgE/Mock/DNP-HSA	IgE/PD98059/PBS	IgE/PD98059/DNP-HSA
**IL-4**	0.958292822	2.171957062	1.259838249	1.847101772
	1.026162363	1.954131907	1.324674274	1.476353637
	1.015544815	2.229716467	1.216091071	1.607293248
**IL4R**	1.009062352	2.450039909	1.056399482	1.4045213
	1.019562251	2.585032769	1.409702244	1.130566891
	0.971375396	2.898582435	1.070174167	1.252297959

Fig.6B RBL2H3_Roc-A

Sample / Target gene	IgE/Mock/PBS	IgE/Mock/DNP-HSA	IgE/Roc-A/PBS	IgE/Roc-A/DNP-HSA
**c-JUN**	1.004427671	3.900514207	1.099799612	1.448467283
	1.025409946	5.533831001	1.432498281	1.467938288
	0.970162383	4.873529993	1.197404557	1.218146218

Fig.7A RBL2H3_IL-4 neutralizing antibody

Sample / Target gene	IgE/IgG/PBS	IgE/IgG/DNA-HSA	IgE/nIL-4/PBS	IgE/nIL-4/DNP-HSA
**IL-4**	1.062094377	3.156832245	0.562621055	0.519773414
	1.11462972	3.531128221	0.651504136	0.600207564
	0.823275902	3.305015122	0.863375466	0.854929261
**IL-4R**	1.466497289	2.293230258	0.722410449	1.514603758
	0.610234669	3.593053147	0.594674001	1.179049046
	0.923268043	2.922668563	0.947771039	1.240963784

Fig.7F RBL2H3_recombinant IL-4

Sample / Target gene	Mock	25ng/ml rIL-4	50ng/ml rIL-4	100ng/ml rIL-4
**CXCL1**	1.059684003	1.466668095	3.363971961	8.297380319
	0.967734493	1.43714677	3.570122261	8.861285423
	0.972581504	1.455149116	3.175543148	7.919516418

Fig.8C PCA_IL-4 neutralizing antibody

Sample / Target gene	IgE/IgG/PBS	IgE/IgG/DNA-HSA	IgE/nIL-4/PBS	IgE/nIL-4/DNP-HSA
**IL-4**	1.288322585	5.704846469	1.428892252	2.12603412
	0.721734997	3.898382007	1.212385101	2.446947331
	0.989942418	4.089513196	1.163405484	1.456119727

Fig.9A RBL2H3_Rocaglamide A

Target gene \ Sample	IgE/Mock/PBS	IgE/Mock/DNP-HSA	IgE/Roc-A/PBS	IgE/Roc-A/DNP-HSA
**miR-449a**	0.950726184	0.34076939	0.915963953	0.882764486
	1.009380667	0.354221197	1.037395707	0.863523234
	1.039893148	0.378034236	1.128933357	0.92391835
**miR-34a-5p**	0.948093562	0.485945369	1.056209457	0.861239106
	1.057679127	0.388018449	1.074047841	0.915003985
	0.99422731	0.433412214	1.051052664	0.874391681

Fig.9B RBL2H3_miR-34a mimic

Target gene \ Sample	Ctrl.mimic/IgE/PBS	Ctrl.mimic/IgE/DNP-HSA	miR-34a mimic/IgE/PBS	miR-34a mimic/IgE/DNP-HSA
**miR-34a-5p**	1.061346115	0.592465494	22.68657536	3.093398964
	1.019415403	0.506170395	21.71399973	2.893666203
	0.919238482	0.518210302	20.49573818	2.934996208

Fig.9D RBL2H3_siIL-4R

Target gene \ Sample	IgE/siCtrl./PBS	IgE/siCtrl./DNP-HSA	IgE/siIL-4R/PBS	IgE/siIL-4R/DNP-HSA
**miR-34a-5p**	0.879305022	0.2542645	1.598525998	1.23498721
	0.924028254	0.277140189	1.751801673	0.960242327
	1.196666725	0.356166856	1.781651749	1.02777561

Fig.12B PCA_1536801

Target gene \ Sample	IgE/Mock/PBS	IgE/Mock/DNP-HSA	IgE/1536801/PBS	IgE/1536801/DNP-HSA
**IL-4**	0.381089548	1.361446984	1.275222492	1.222809951
	1.131056812	4.412772742	1.417790468	0.472085145
	1.48785364	3.859234291	1.455233992	1.128030562
**IL4R**	1.128723206	16.98438835	0.666774295	1.379539013
	0.918152592	29.05667001	2.951736693	1.825756763
	0.953124202	41.34234693	1.66625702	0.777761763
**c-JUN**	0.953470942	6.270482898	0.720582639	2.10857731
	0.965849881	6.532389876	0.857527191	1.831392262
	1.080679177	6.864295577	1.152504042	1.916669129

Fig.13B PSA_1536801

Target gene \ Sample	IgE/Mock/PBS	IgE/Mock/DNP-HSA	IgE/1536801/PBS	IgE/1536801/DNP-HSA
**IL-4**	0.506751578	3.620752187	0.322251751	1.069536965
	1.504016123	5.867858517	0.497641565	0.31861403
	0.989232299	5.131794028	0.297274179	0.61025686
**IL-4R**	1.128723206	8.492194176	0.136701173	0.21027644
	0.918152592	7.264167502	0.180917748	0.930870745
	0.953124202	10.33558673	0.154139817	0.525477058

Supple Fig.2C RBL2H3_siC-JUN

Target gene \ Sample	IgE/Mock/PBS	IgE/Mock/DNP-HSA	IgE/PD98059/PBS	IgE/PD98059/DNP-HSA
**IL-4**	1.011407297	2.08278321	0.199351791	0.418327396
	0.977574444	1.970758746	0.189648441	0.407058514
	1.01101826	1.937048769	0.185373534	0.403194141
**IL4R**	1.045466967	2.692301847	0.372884525	1.066453901
	0.978816291	2.552331476	0.357793254	1.078541546
	0.975716742	2.545123896	0.319968622	1.022465078
**c-JUN**	0.929370981	2.150500886	0.123446001	0.113728435
	1.000823533	1.712682597	0.134574332	0.178765032
	1.069805486	1.45944611	0.30924233	0.133417622

Supple Fig.S3B PSA_siIL-4R

Target gene \ Sample	IgE/siCtrl./PBS	IgE/siCtrl./DNP-HSA	IgE/siIL-4R/PBS	IgE/siIL-4R/DNP-HSA
**IL-4R**	0.980567573	4.127208677	1.199708774	1.168377716
	1.018150702	4.484045798	1.218408836	1.161306028
	1.001281725	4.40484103	1.249858144	1.43915426

β-hexosaminidase activity

Fig.1B RBL2H3_Roc-A

IgE/Mock	IgE/DNP-HSA	IgE/Roc-A 1hr/DNP-HSA	IgE/Roc-A 2hr/DNP-HSA	IgE/Roc-A 4hr/DNP-HSA	IgE/Roc-A 6hr/DNP-HSA
97.56097561	202.4390244	137.804878	125.6097561	114.6341463	97.56097561
100	196.3414634	141.4634146	128.0487805	102.4390244	104.8780488
102.4390244	193.902439	143.902439	128.0487805	103.6585366	104.8780488

Fig.2B PCA_Roc-A

IgE/Mock	IgE/DNP-HSA	IgERoc-A//DNP-HSA
100.4464286	179.4642857	123.2142857
101.7857143	168.75	128.5714286
97.76785714	170.0892857	125.8928571

Fig.7B RBL2H3_nIL4

IgE/IgG/PBS	IgE/IgG/DNP-HSA	IgE/nIL-4 10ng/ml/DNP-HSA	IgE/nIL-4 100ng/ml/DNP-HSA	IgE/nIL-4 1μg/ml/DNP-HSA	IgE/nIL-4 10μg/ml/DNP-HSA
95.23809524	449.4047619	285.7142857	317.2619048	241.6666667	190.4761905
100.5952381	415.4761905	281.547619	337.9761905	276.1904762	208.3333333
104.1666667	560.1190476	296.4285714	345.8333333	251.7857143	208.3333333

Fig.7E RBL2H3_siIL-4R

siCtrl./IgE/PBS	siCtrl./IgE/DNP-HSA	10nM siIL-4R/IgE/DNP-HSA	20nM siIL-4R/IgE/DNP-HSA	40nM siIL-4R/IgE/DNP-HSA	80nM siIL-4R/IgE/DNP-HSA
101.4893617	333.1914894	202.3404255	133.4042553	132.7659574	136.5957447
100.212766	329.3617021	206.8085106	137.2340426	141.0638298	145.5319149
98.29787234	331.2765957	209.3617021	140.4255319	140.4255319	155.106383

Fig.9C RBL2H3_miR-34a mimic

Ctrl.mimic/IgE/PBS	Ctrl.mimic/IgE/DNP-HS[illegible]	[illegible]M miR-34a mimic/IgE/DNP-[illegible]	[illegible]nM miR-34a mimic/IgE/DNP-H[illegible]	[illegible]nM miR-34a mimic/IgE/DNP-H[illegible]	[illegible]0nM miR-34a mimic/IgE/DNP-HSA
101.3513514	489.7297297	376.2162162	347.027027	254.5945946	132.1621622
100.5405405	499.4594595	374.5945946	334.0540541	305.6756757	116.7567568
98.10810811	490.5405405	380.2702703	361.6216216	331.6216216	111.8918919

Fig.11A RBL2H3_Chemical 1

IgE/Mock	IgE/DNP-HSA	IgE/DNP-HSA/ chem._0.01μM	IgE/DNP-HSA/ chem._0.1μM	IgE/DNP-HSA/ chem._1μM	IgE/DNP-HSA/ chem._10μM
96.63461538	302.8846154	300	294.2307692	248.0769231	181.7307692
106.7307692	317.3076923	310.0961538	297.1153846	255.2884615	170.1923077
96.63461538	320.1923077	314.4230769	311.5384615	248.0769231	160.0961538

Fig.11B RBL2H3_Chemical 2

IgE/Mock	IgE/DNP-HSA	IgE/DNP-HSA 0.01μM	IgE/DNP-HSA 0.1μM	IgE/DNP-HSA 1μM	IgE/DNP-HSA 10μM
97.73755656	298.6425339	294.5701357	282.3529412	215.8371041	154.7511312
104.5248869	293.2126697	304.0723982	280.9954751	226.6968326	173.7556561
97.73755656	297.2850679	309.5022624	279.638009	222.6244344	194.1176471

Fig.11C RBL2H3_Chemical 3

IgE/Mock	IgE/DNP-HSA	IgE/DNP-HSA 0.01μM	IgE/DNP-HSA 0.1μM	IgE/DNP-HSA 1μM	IgE/DNP-HSA 10μM
80.29739777	210.7806691	200.7434944	187.3605948	200.7434944	180.669145
108.1784387	199.6282528	228.6245353	195.1672862	201.8587361	198.5130112
111.5241636	214.1263941	195.1672862	184.0148699	208.5501859	229.739777

Evansblue

Fig.2A PCA_Roc-A

IgE/Mock	IgE/PBS/DNP-HSA	IgE/Roc-A/DNP-HSA
4.56868	20.73472	13.96246
4.78714	20.6619	13.96246
9.0107	32.6772	2.01998
9.22916	32.6772	2.01998
11.5594	48.3335	7.40866
11.99632	48.47914	7.40866

Fig.8A PCA_nIL-4

IgE/IgG/PBS	IgE/IgG/DNP-HSA	IgE/nIL-4/PBS	IgE/nIL-4/DNP-HSA
0.005337	1.0148255	0.042247	0.8081295
0.0034915	1.2325945	0.0440925	1.0000615
0.009028	0.9520785	0.0883845	0.983452
0.060702	0.9705335	0.0810025	0.5128495

Fig.8D PCA_siIL-4R

siCtrl./IgE/PBS	siCtrl./IgE/DNP-HSA	siIL-4R/IgE/PBS	siIL-4R/IgE/DNP-HSA
0.0129185	0.11534375	0.0166095	0.10427075
0.01199575	0.276825	0.003691	0.1347215
0.00645925	0.428156	0.02491425	0.158713
0.0129185	0.424465	0.01568675	0.0941205

Fig.12A PCA_1536801

IgE/Mock	IgE/DNP-HSA	IgE/1536801/PBS	IgE/1536801/DNP-HSA
0.46414325	0.930132	0.1494855	0.46598875
0.31465775	0.88860825	0.64869325	0.4078555
0.0756655	1.3748975	0.25929275	0.1937775
0.01753225	0.871076	0.26298375	0.22146

IL-4 ELISA

Fig.4B RBL2H3_Roc-A

IgE/Mock	/IgE/DNP-HSA	IgE/Roc-A/PBS	IgE/Roc-A/DNP-HSA
3.43491154	6.681418304	1.124381213	2.047764101
2.047764101	5.752425875	1.124381213	4.36107143

Fig.13C PSA_1536801

IgE/Mock	/IgE/DNP-HSA	IgE/1536801/PBS	IgE/1536801/DNP-HSA
24.71569538	35.09776185	25.65044101	28.93621344
24.71569538	37.0094351	25.1828447	30.82378345
26.58697853	37.0094351	23.78273386	27.0559217
26.58697853	37.0094351	25.65044101	30.3512051

Western Blot

Figure 1A

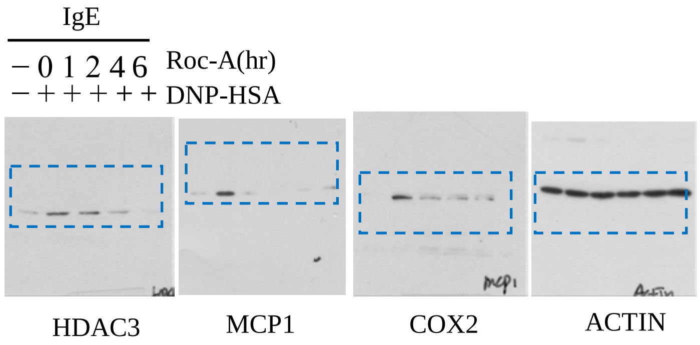

Figure 1C

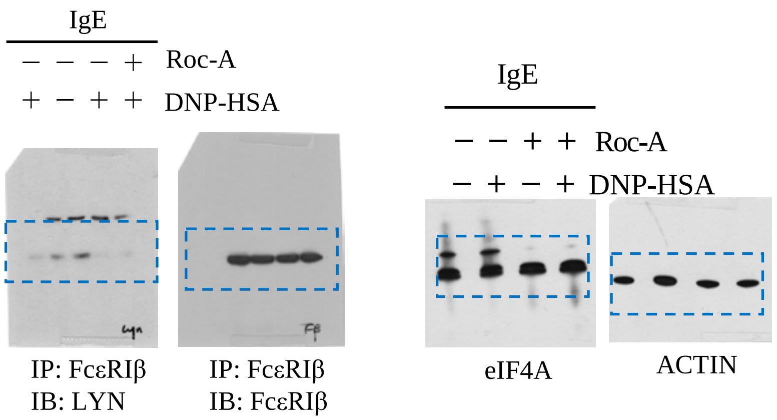

Figure 1D

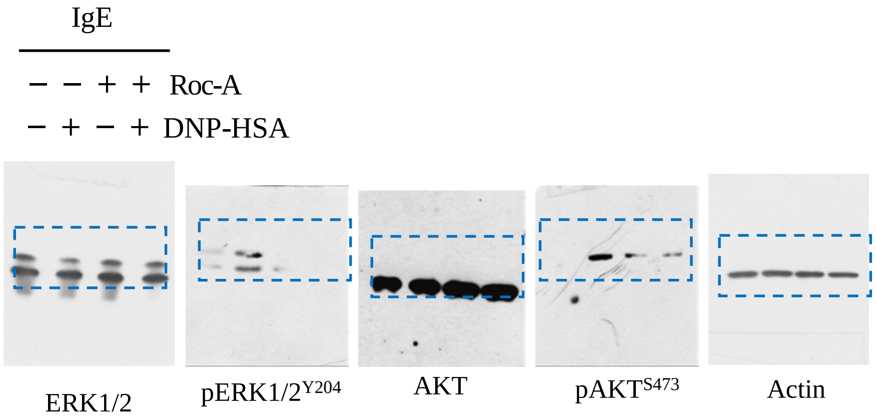

Figure 1D

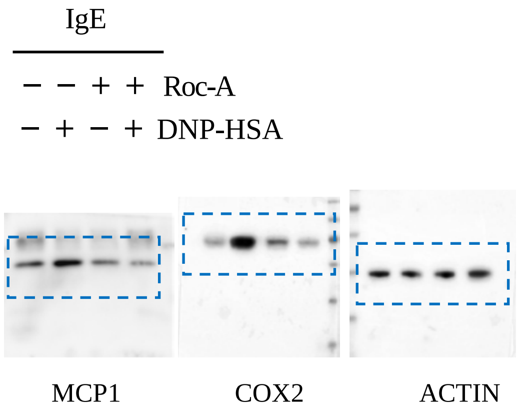

Figure 1F

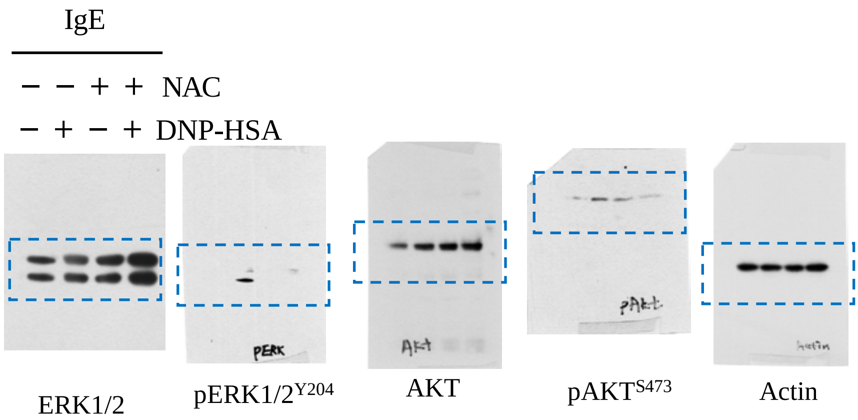

Figure 2C

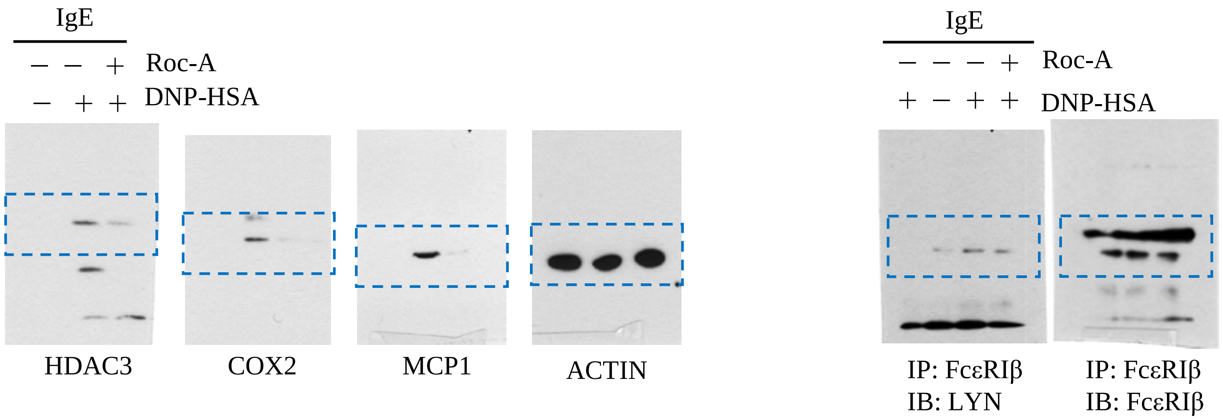

Figure 2E

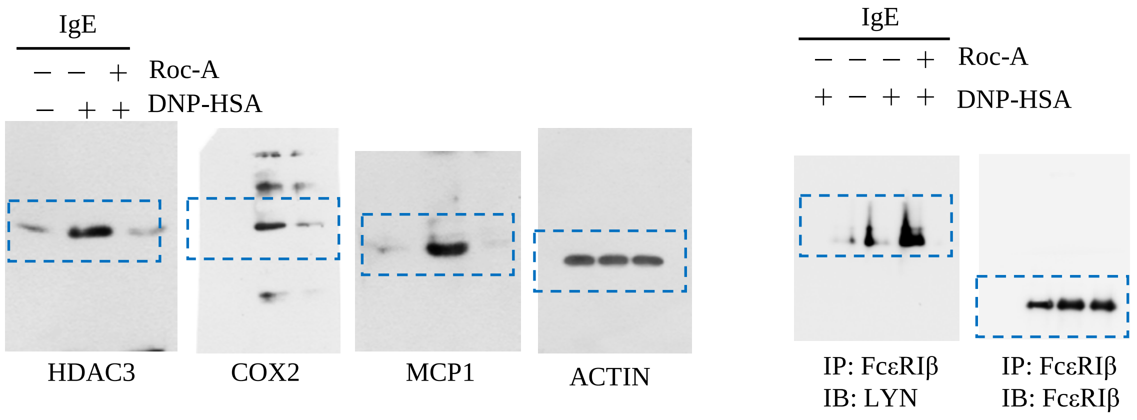

Figure 4C

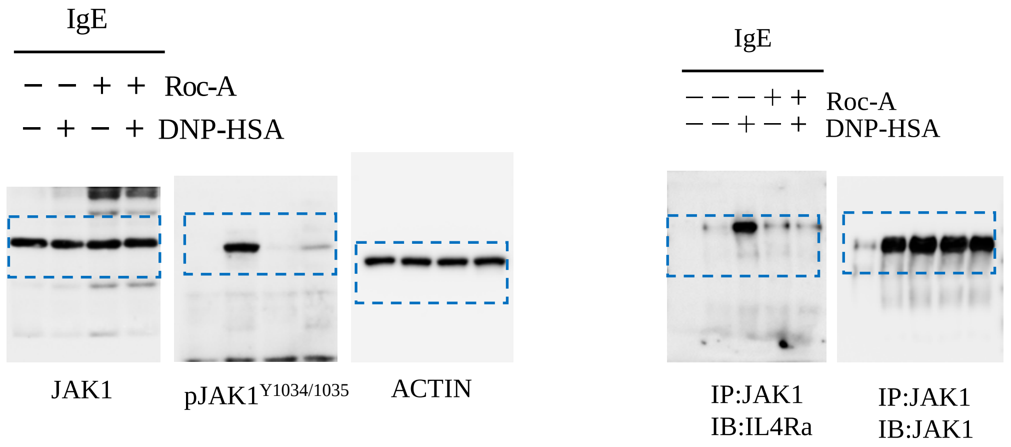

Figure 4E

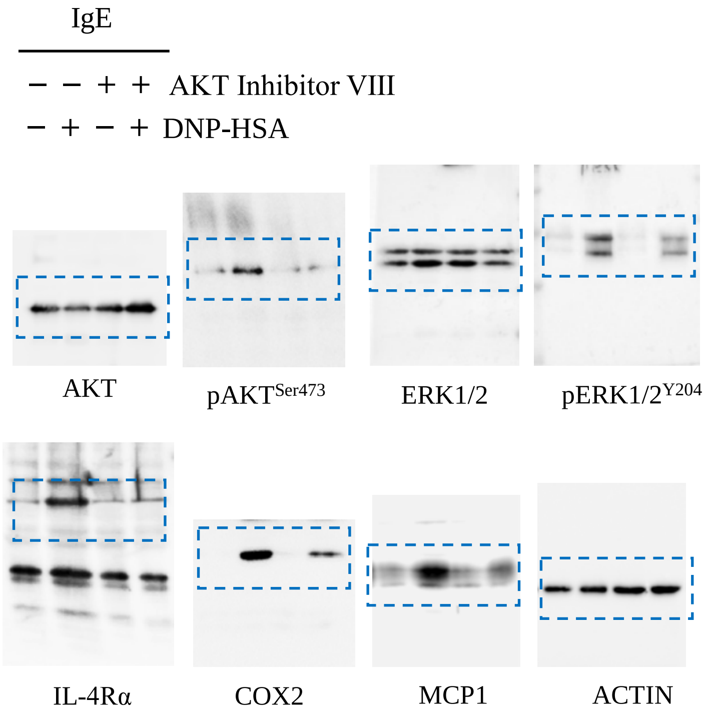

Figure 4G

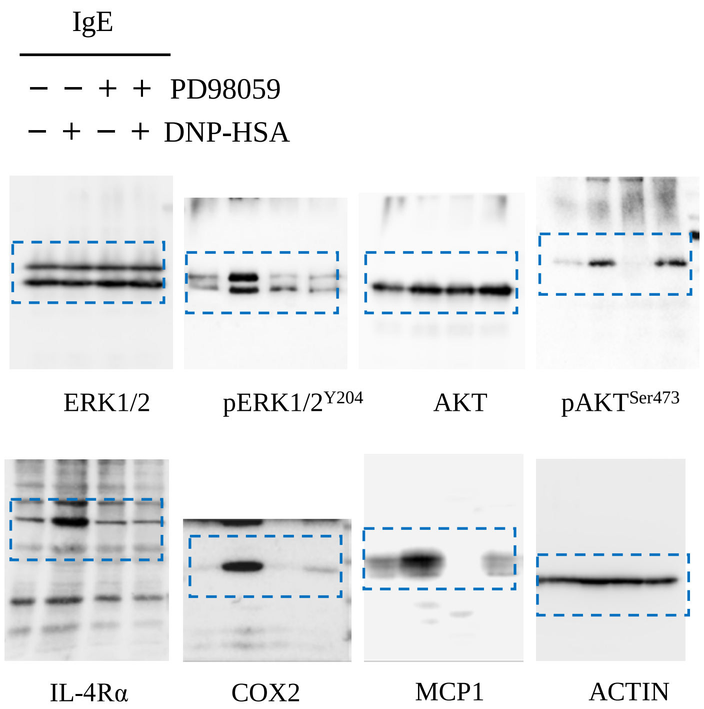

Figure 6B

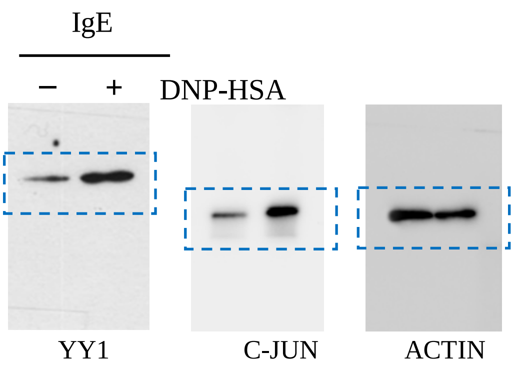

Figure 6B

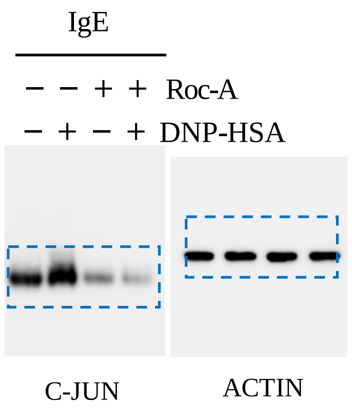

Fig.7C

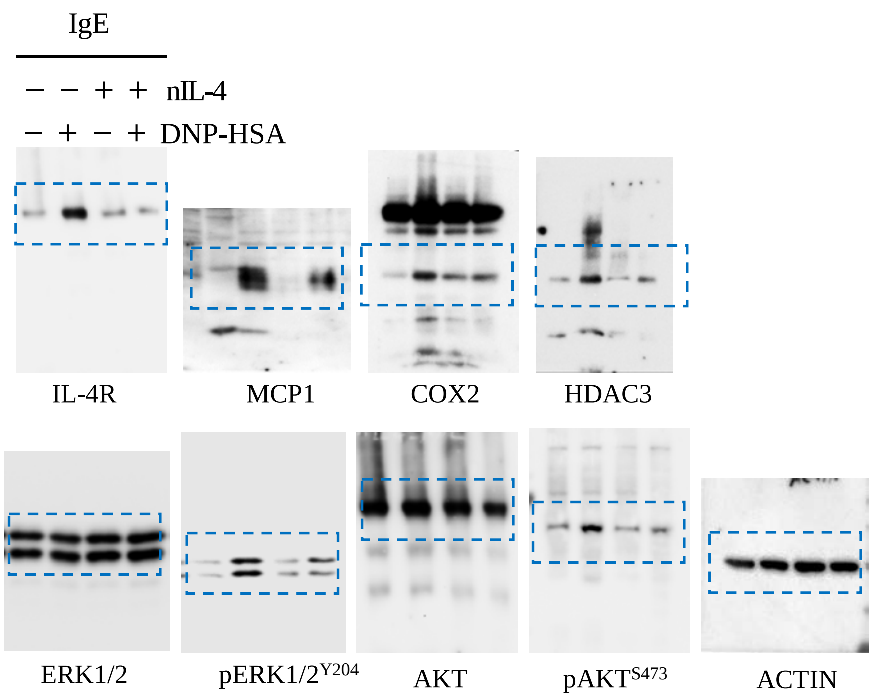
IgE
− − + + nIL-4
− + − + DNP-HSA
IL-4R
MCP1
COX2
HDAC3
ERK1/2
pERK1/2Y204
AKT
pAKTS473
ACTIN

Fig.7D

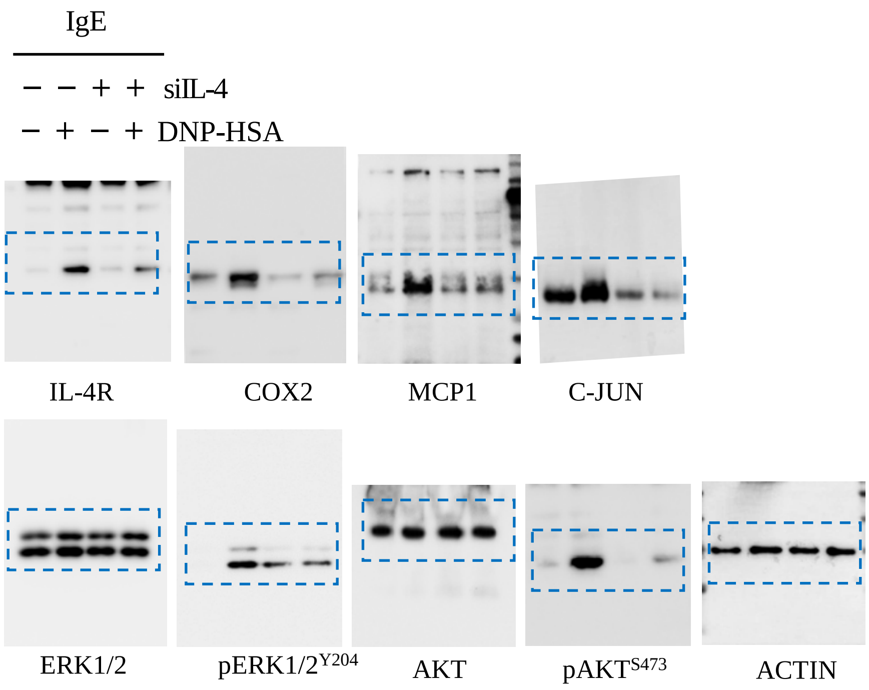
IgE
− − + + siIL-4
− + − + DNP-HSA
IL-4R
COX2
MCP1
C-JUN
ERK1/2
pERK1/2Y204
AKT
pAKTS473
ACTIN

Fig.7F

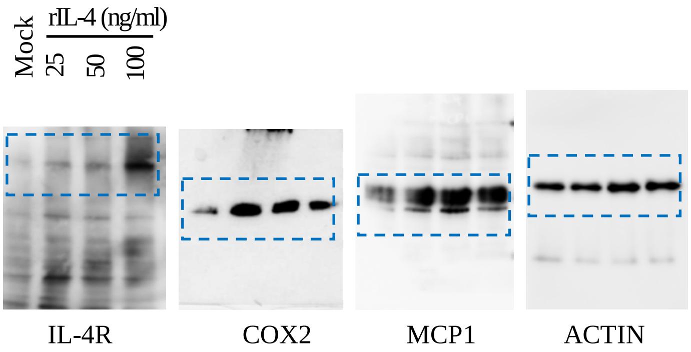

Mock
rIL-4 (ng/ml)
25
50
100
IL-4R
COX2
MCP1
ACTIN

Fig.7G

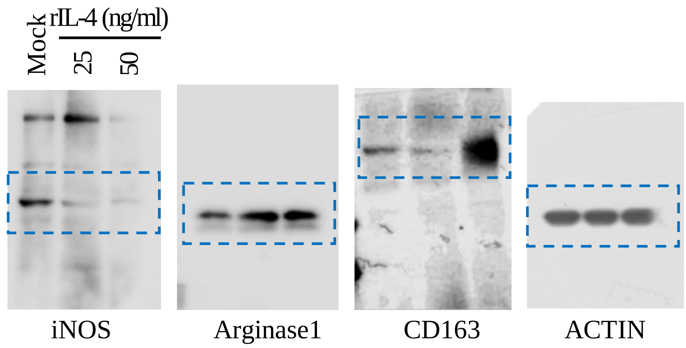

Mock
rIL-4 (ng/ml)
25
50
iNOS
Arginase1
CD163
ACTIN

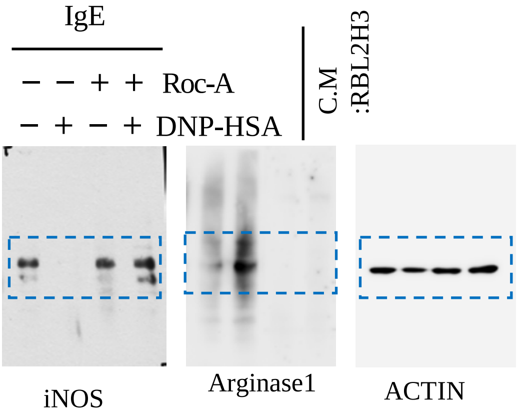

IgE
− − + + Roc-A
− + − + DNP-HSA
C.M
:RBL2H3
iNOS
Arginase1
ACTIN

Fig.8B

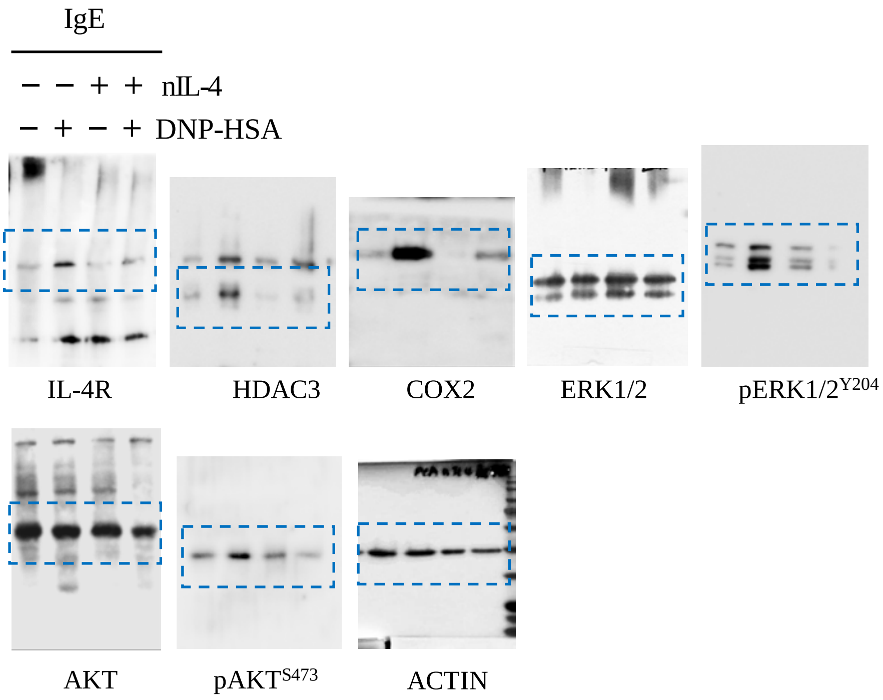

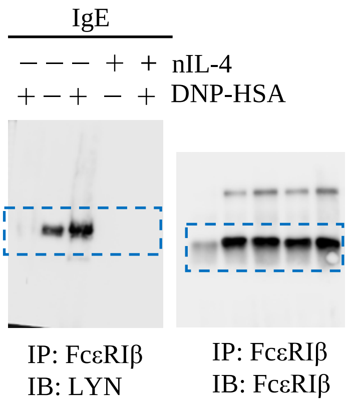

Fig.8D PCA

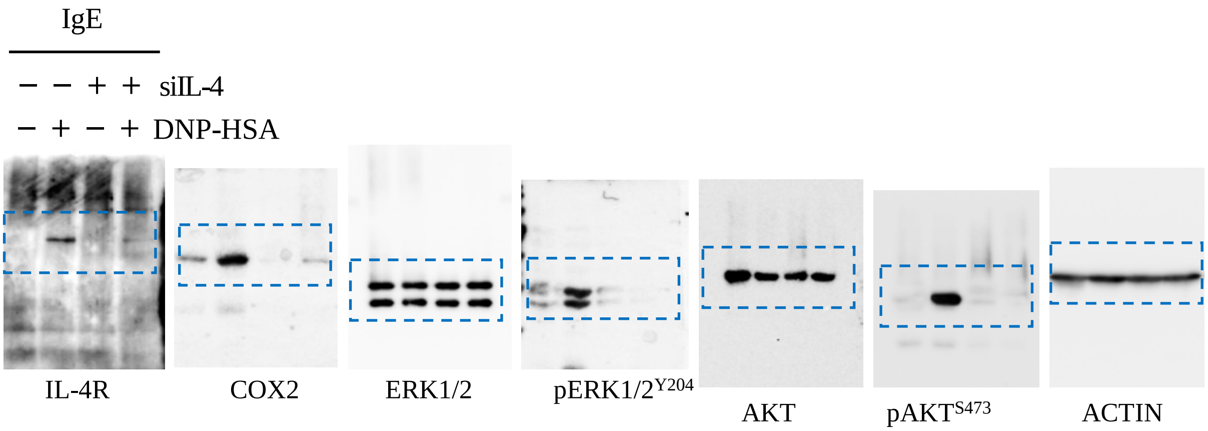

Figure 11B.

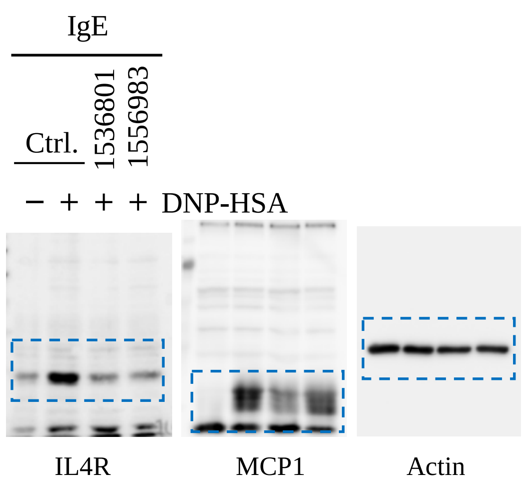

Figure 11C

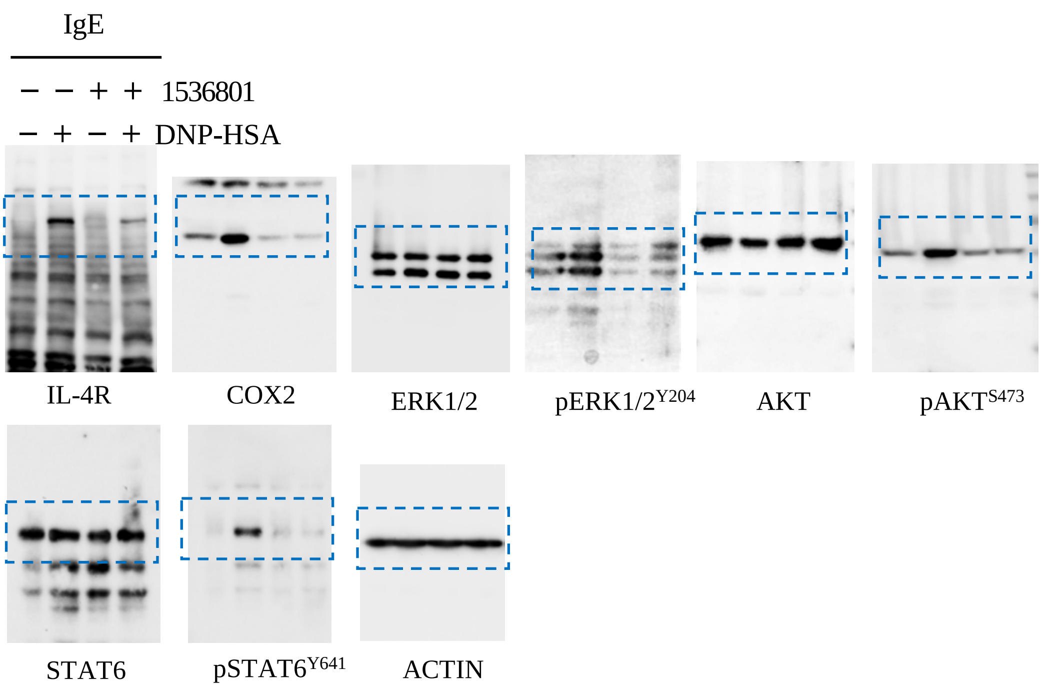

Figure 11C

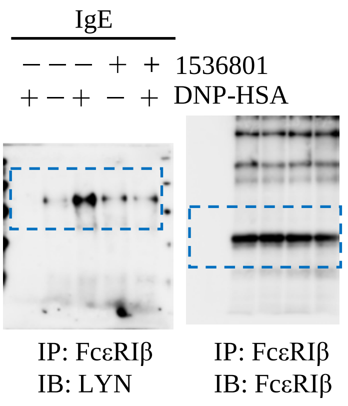

Figure 12C

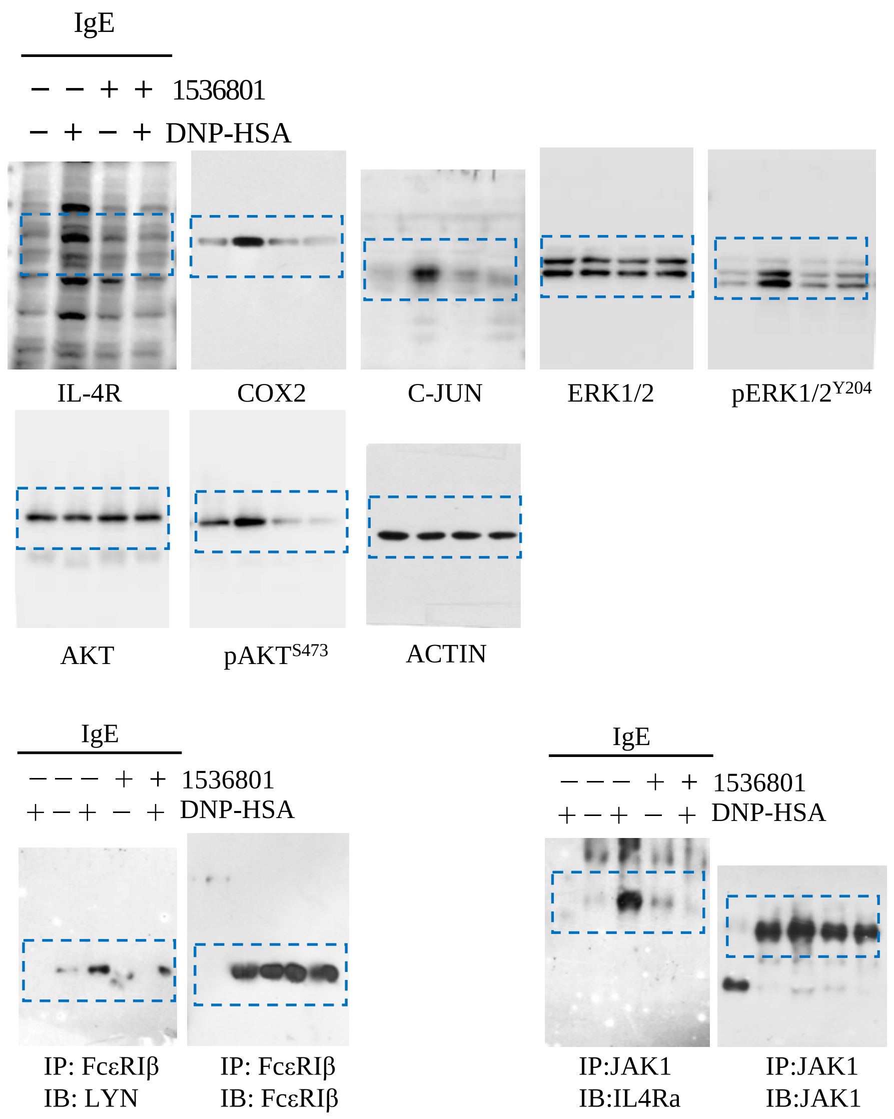

Figure 13C

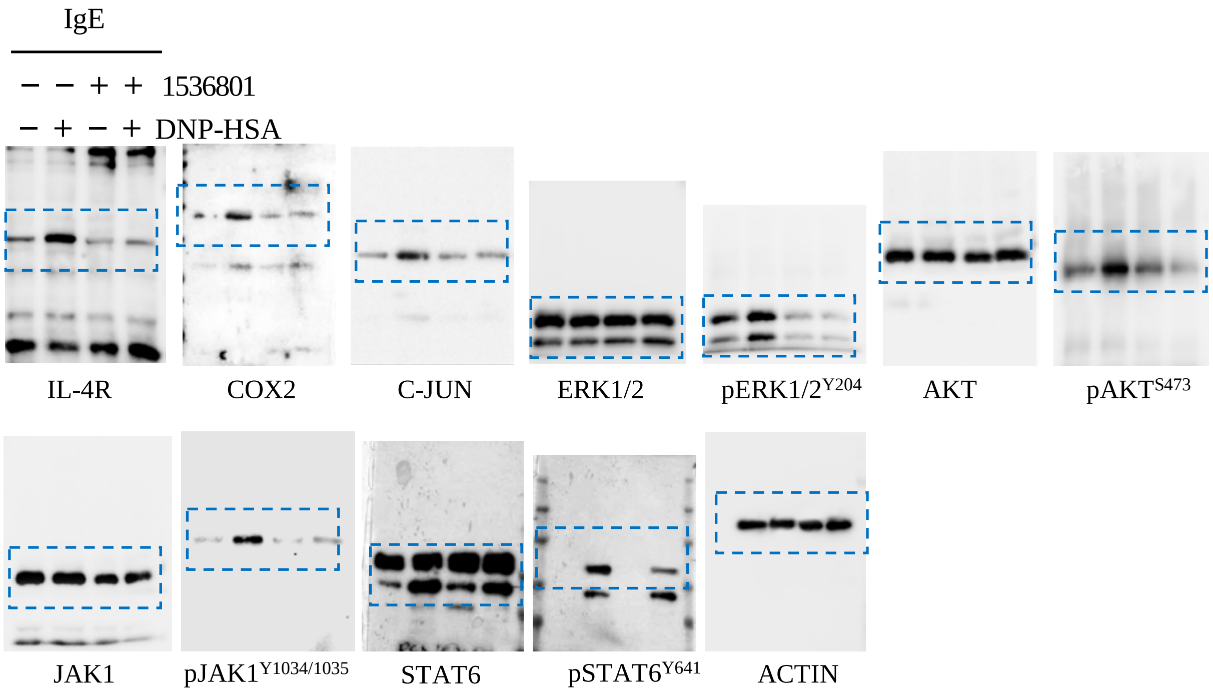

Supple Figure S2D

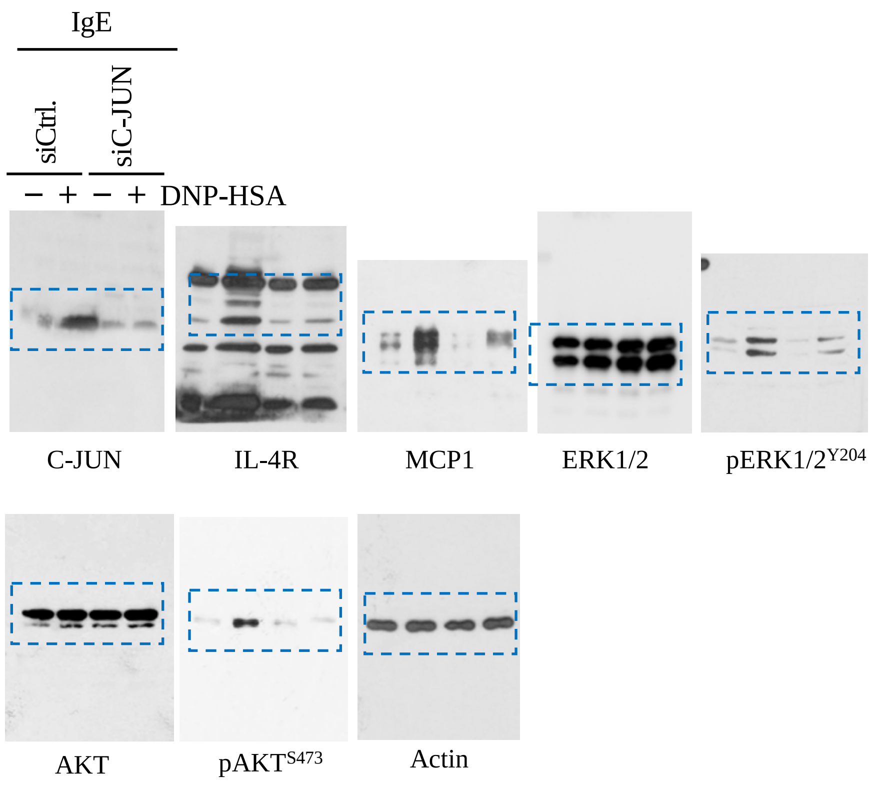

Supple Figure S3C

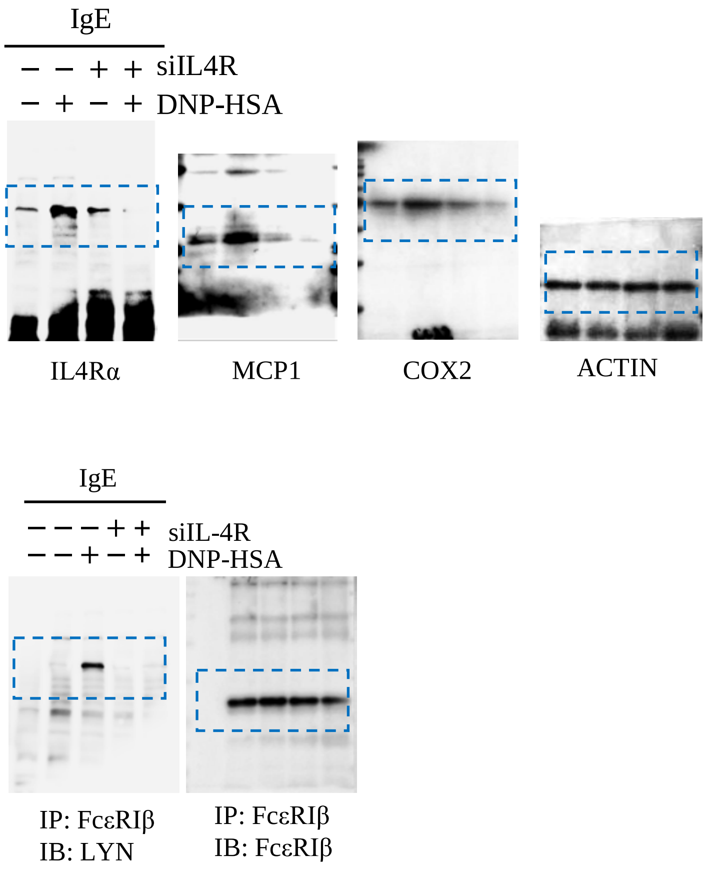

PCA

Figure 8A

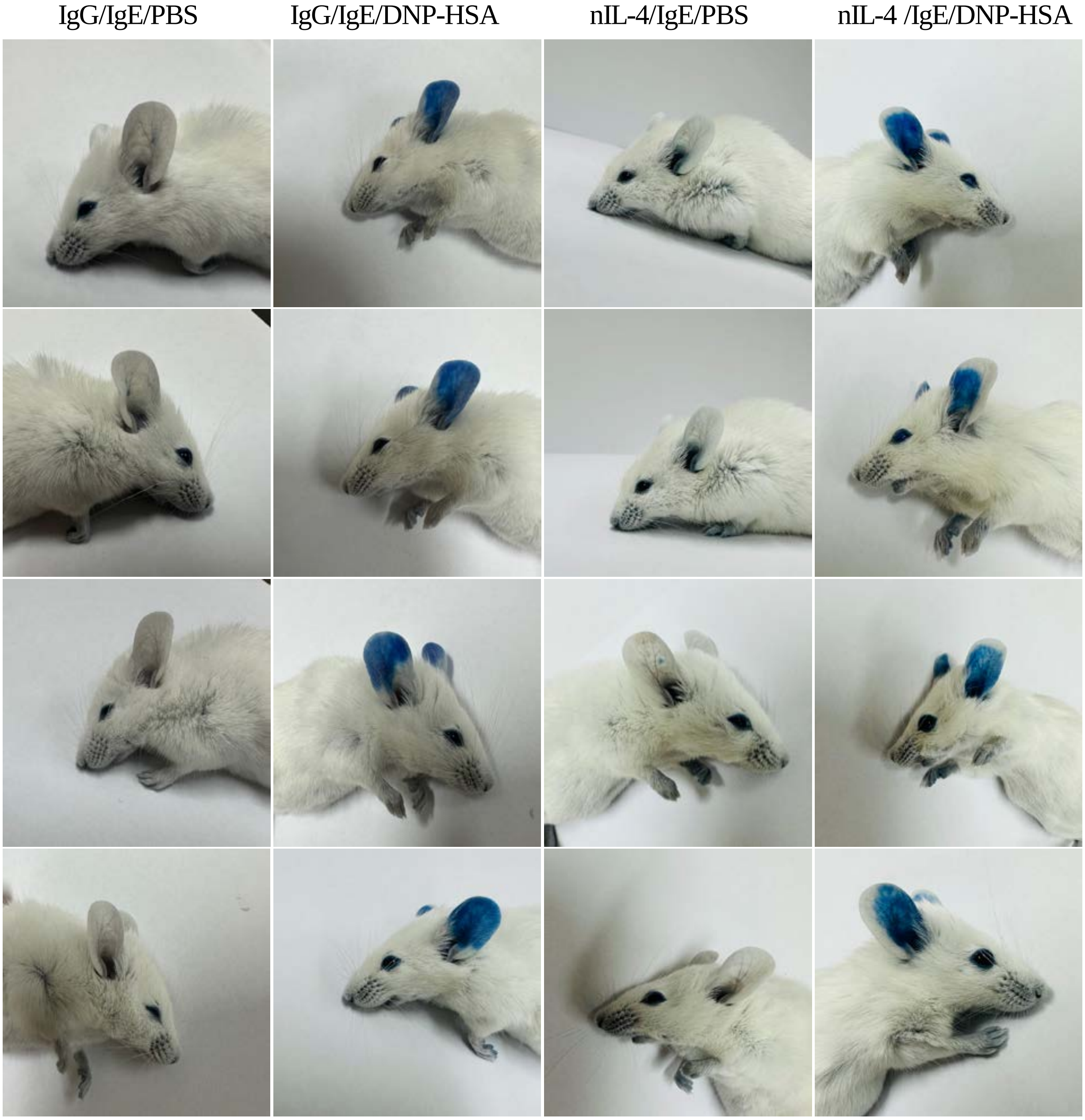

Figure 8D

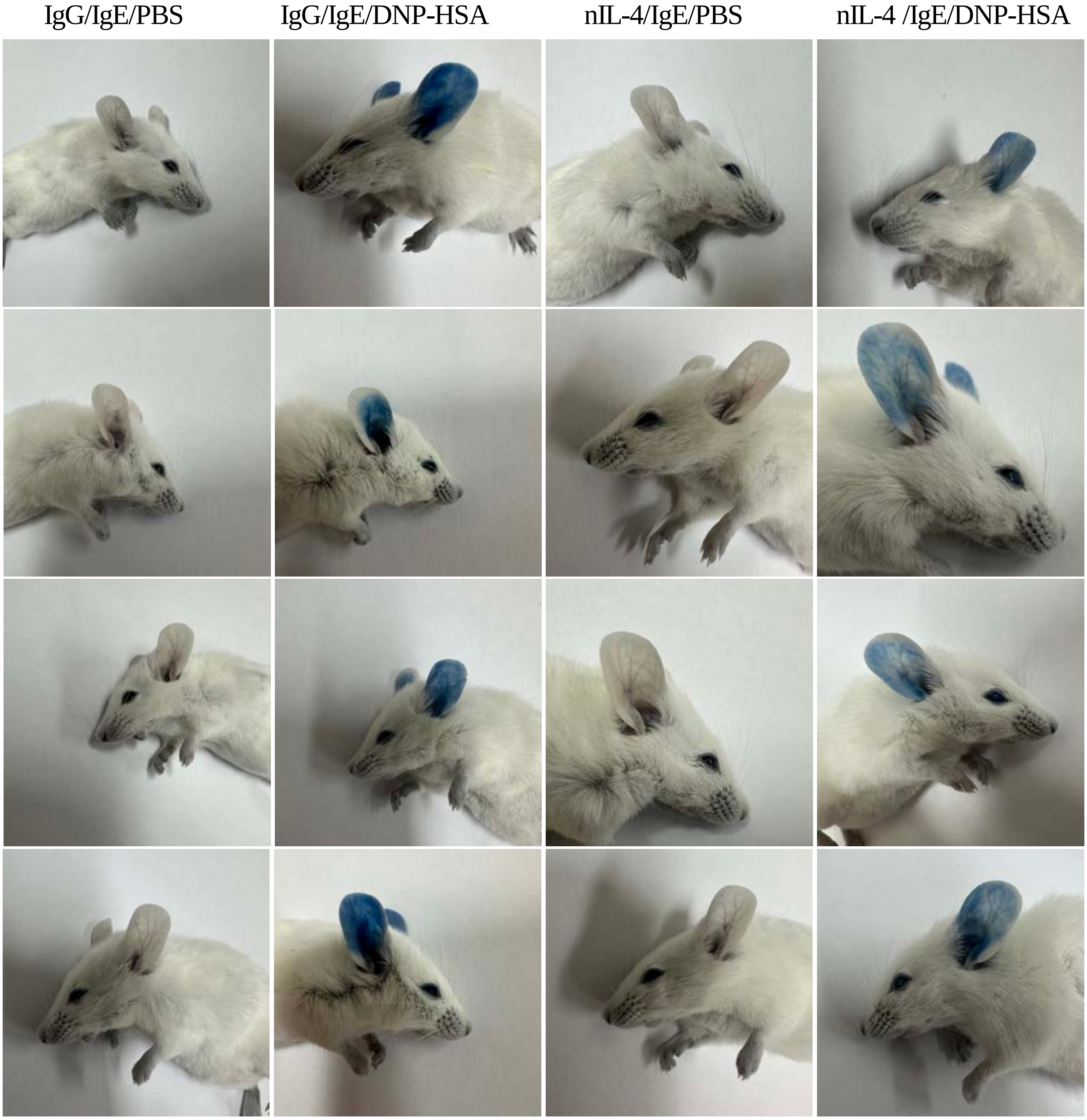

Figure 12A

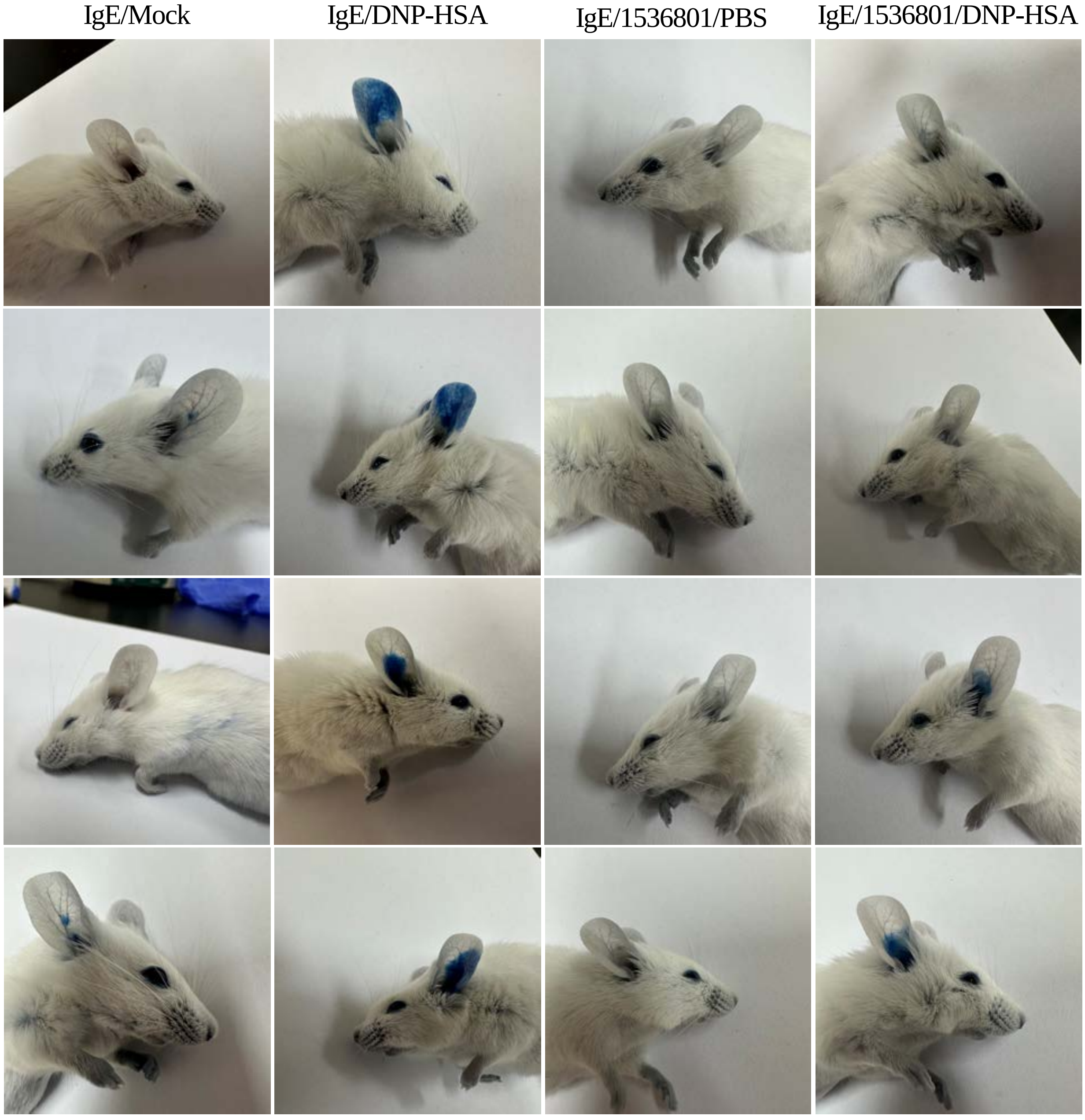

Supplement: Supplementary file 1 [file molecules-30-00840-s001.zip › RAW_data.pdf]
