# Supplementary figures and images for: Rocaglamide Suppresses Allergic Reactions by Regulating IL-4 Receptor Signaling
Source: Molecules. 2025 Feb 11;30(4):840. doi: 10.3390/molecules30040840 (PMC11858170; doi:10.3390/molecules30040840)

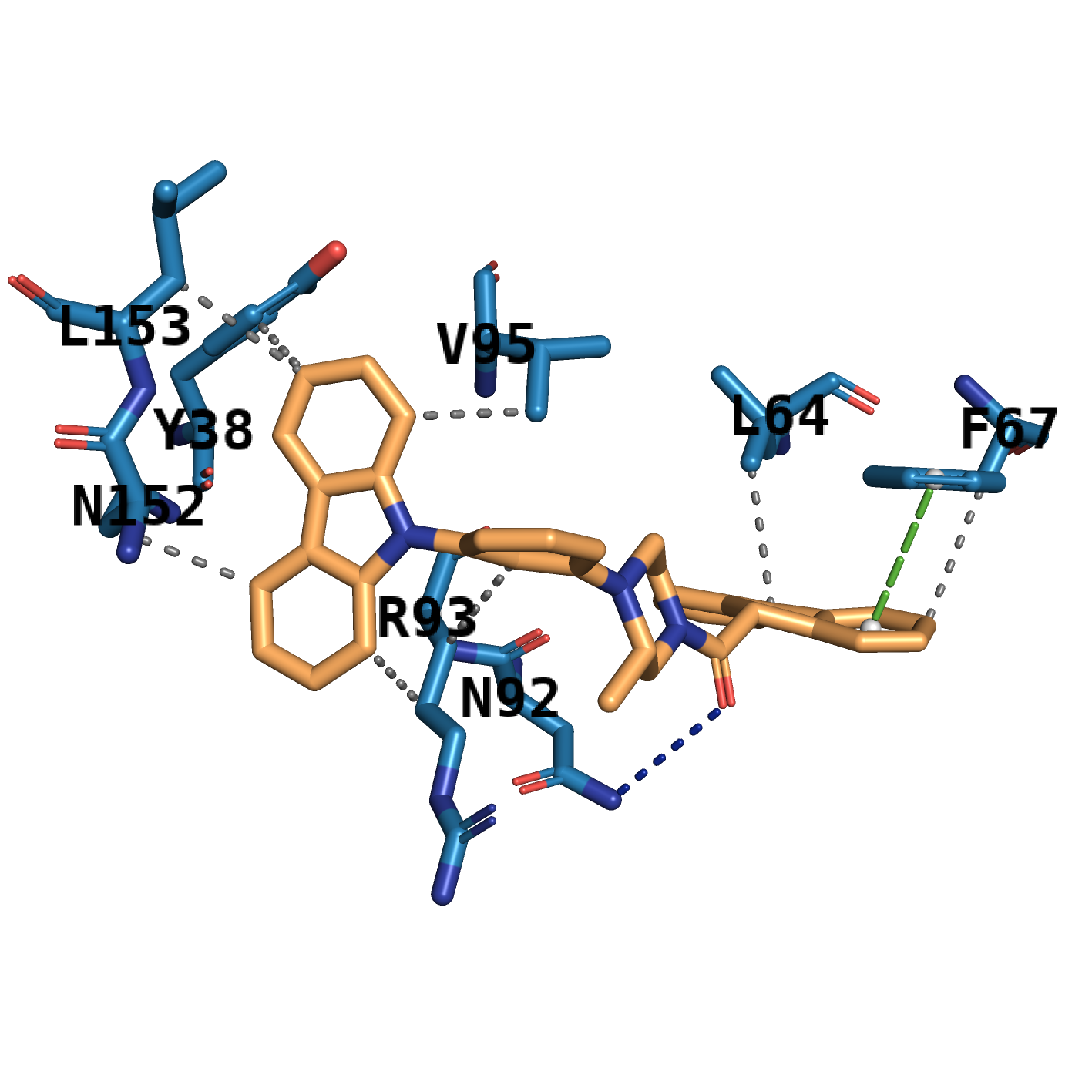

Supplement: Supplementary file 1 [file molecules-30-00840-s001.zip › Figure 10 raw data/1222903.png]

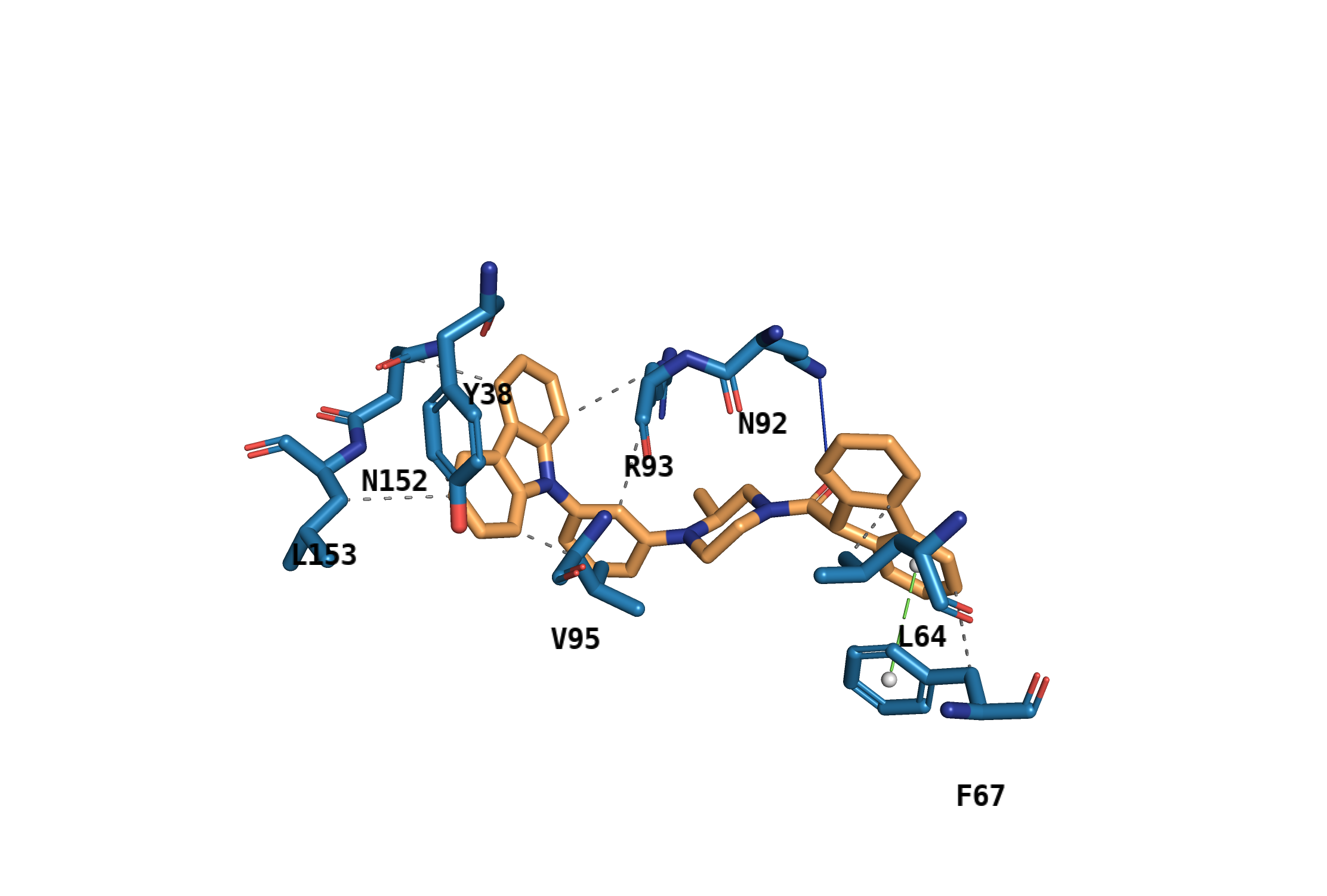

Supplement: Supplementary file 1 [file molecules-30-00840-s001.zip › Figure 10 raw data/1222903-1.png]

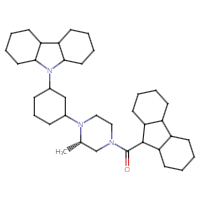

Supplement: Supplementary file 1 [file molecules-30-00840-s001.zip › Figure 10 raw data/1222903-2.png]

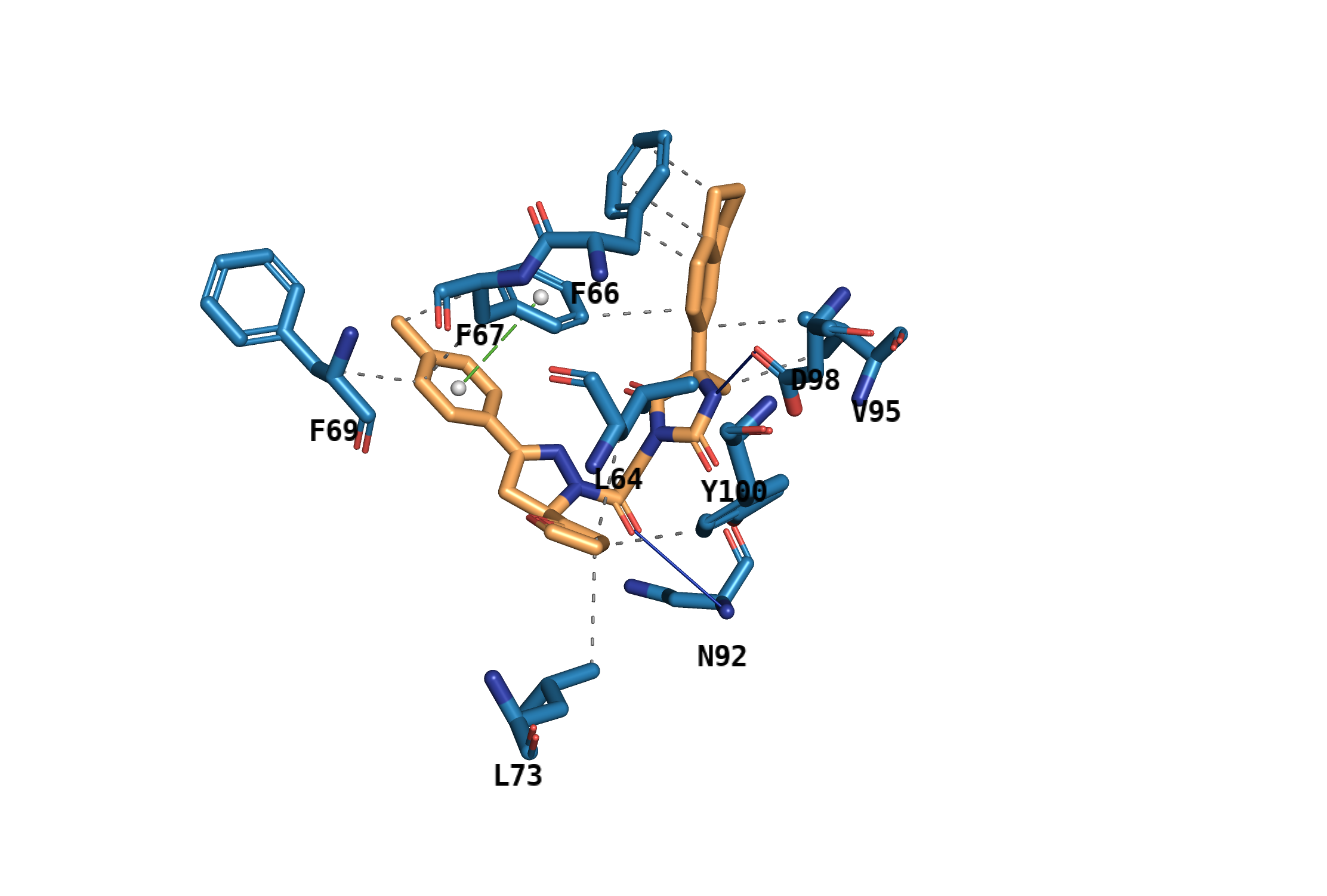

Supplement: Supplementary file 1 [file molecules-30-00840-s001.zip › Figure 10 raw data/1536801-1.png]

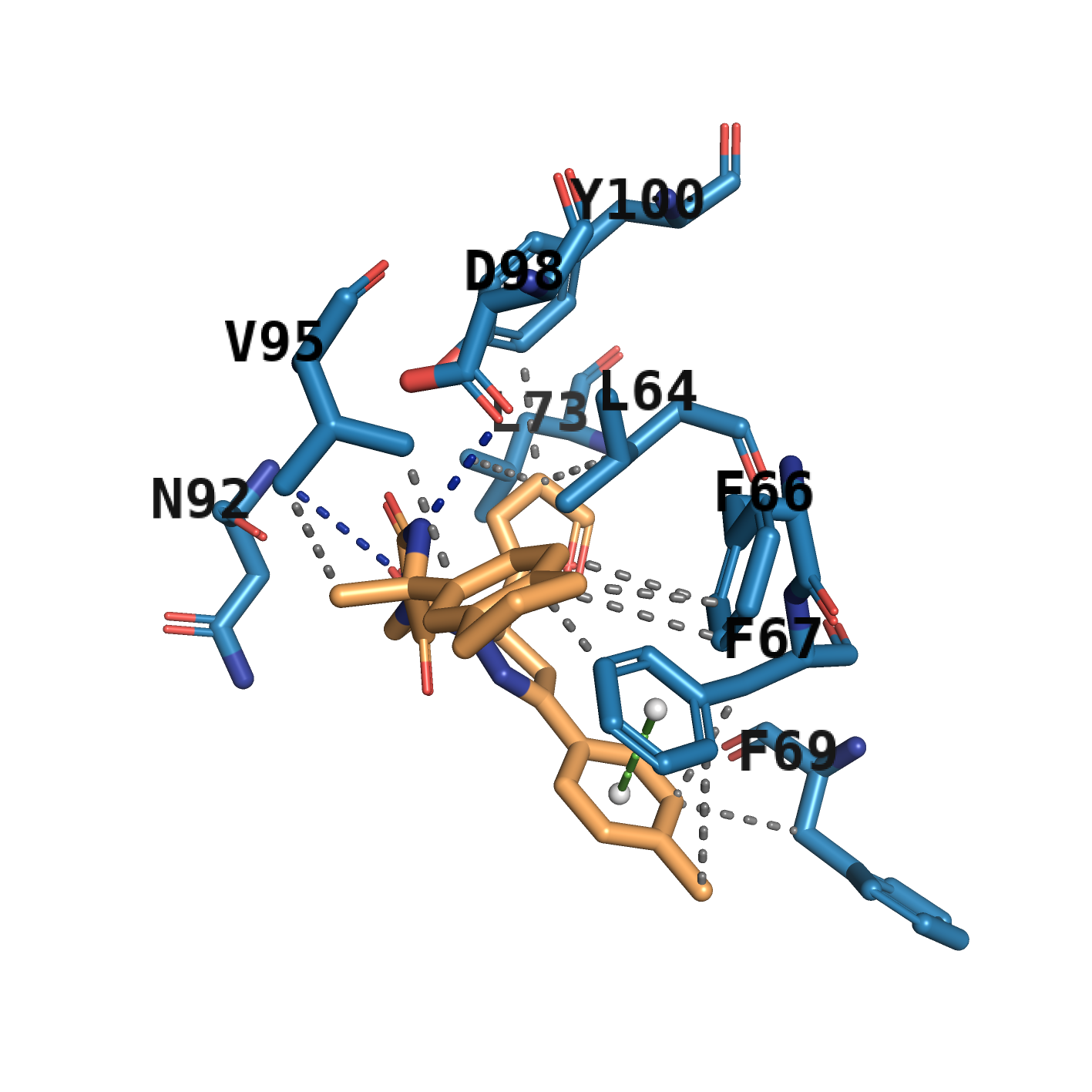

Supplement: Supplementary file 1 [file molecules-30-00840-s001.zip › Figure 10 raw data/1538601.png]

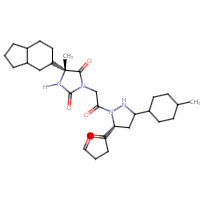

Supplement: Supplementary file 1 [file molecules-30-00840-s001.zip › Figure 10 raw data/1538601-2.png]

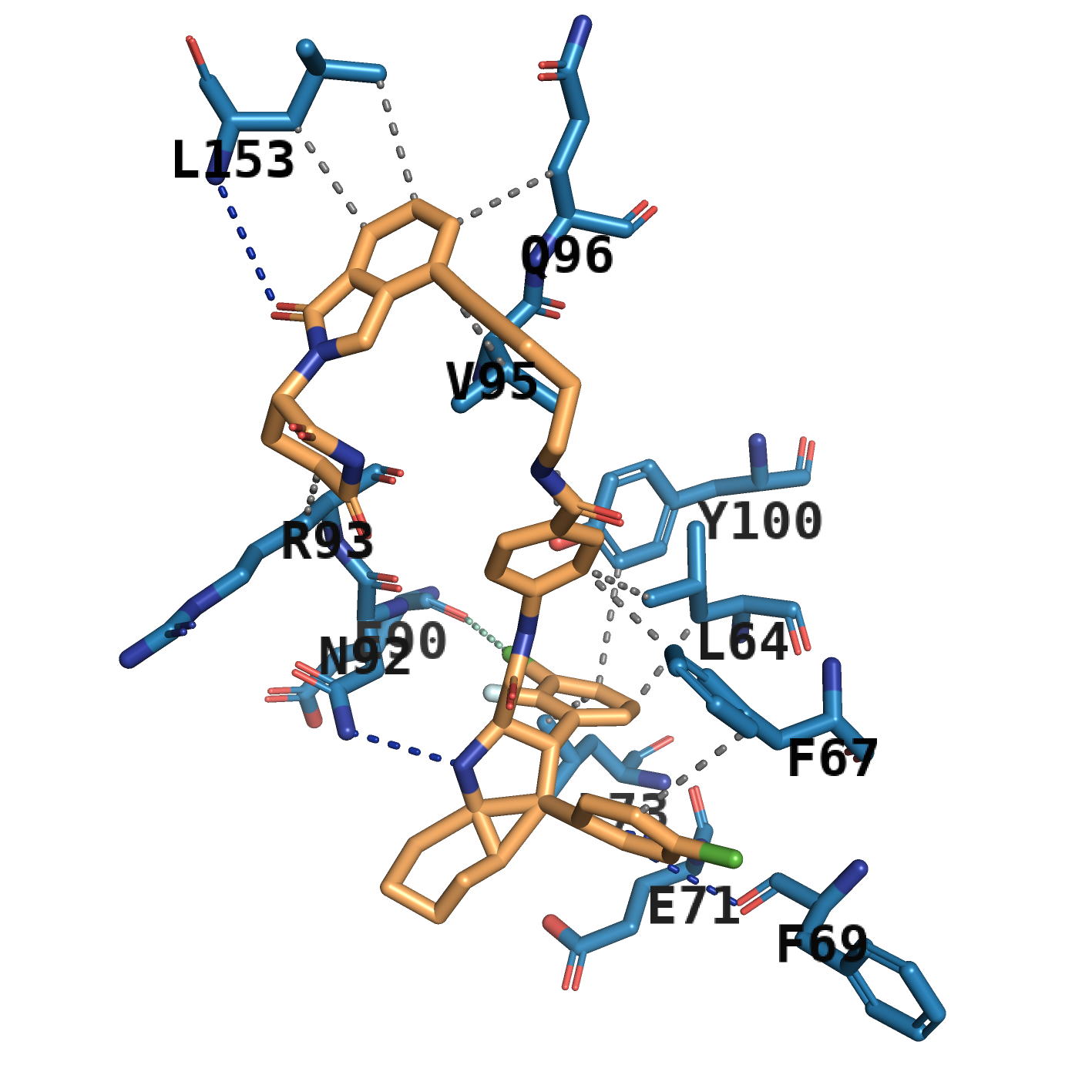

Supplement: Supplementary file 1 [file molecules-30-00840-s001.zip › Figure 10 raw data/1556983.png]

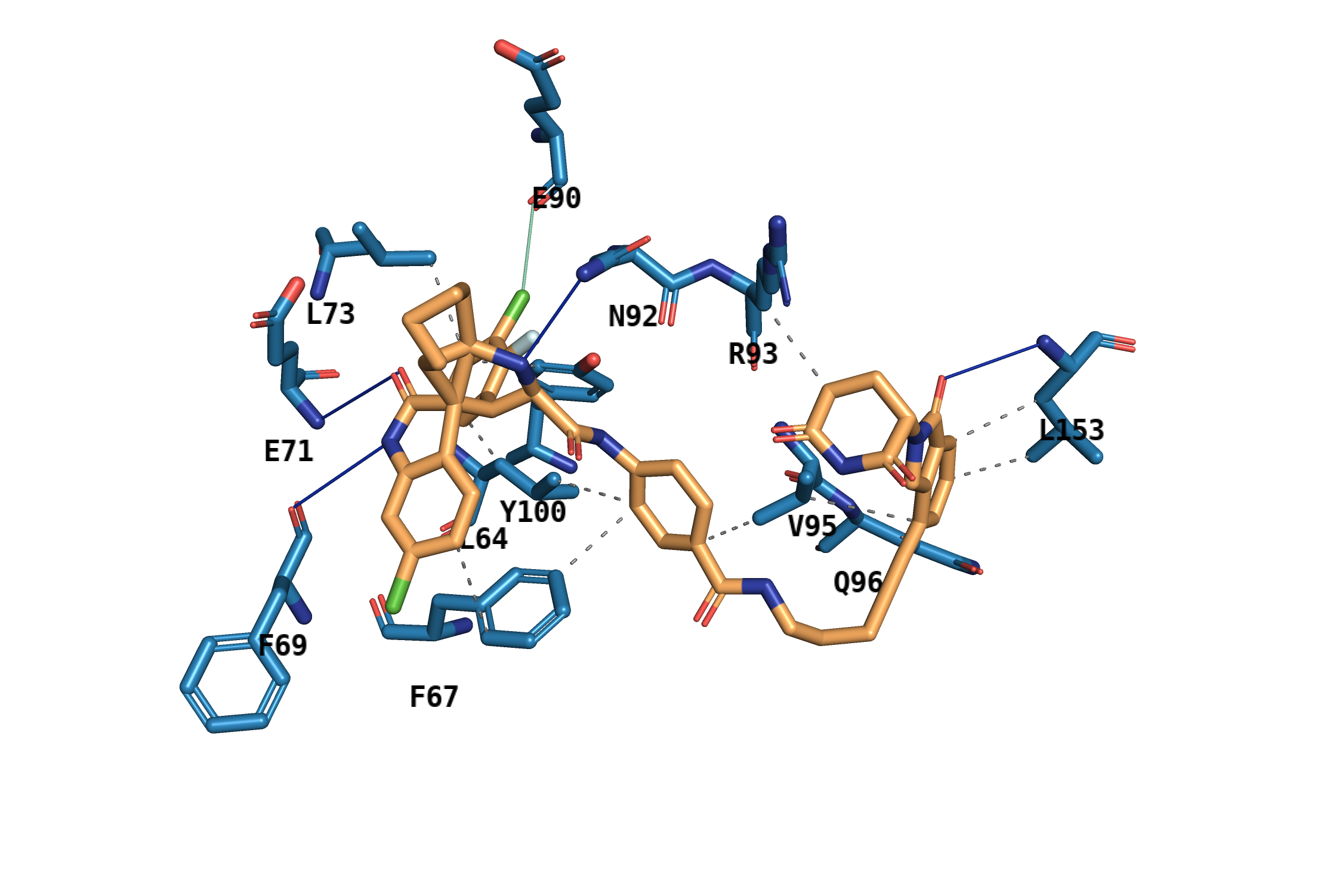

Supplement: Supplementary file 1 [file molecules-30-00840-s001.zip › Figure 10 raw data/1556983-1.png]

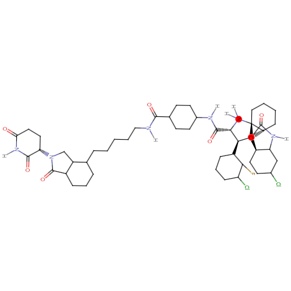

Supplement: Supplementary file 1 [file molecules-30-00840-s001.zip › Figure 10 raw data/1556983-2d.png]

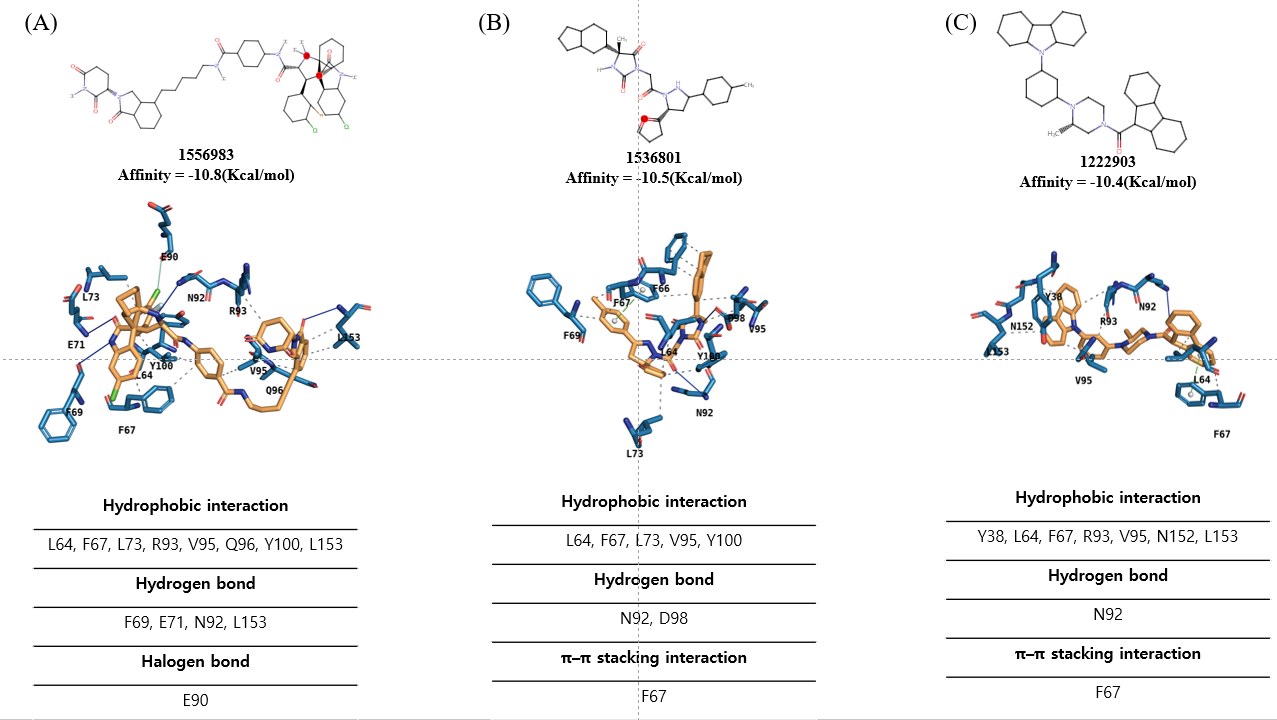

Supplement: Supplementary file 1 [file molecules-30-00840-s001.zip › Figure 10 raw data/final.tif]

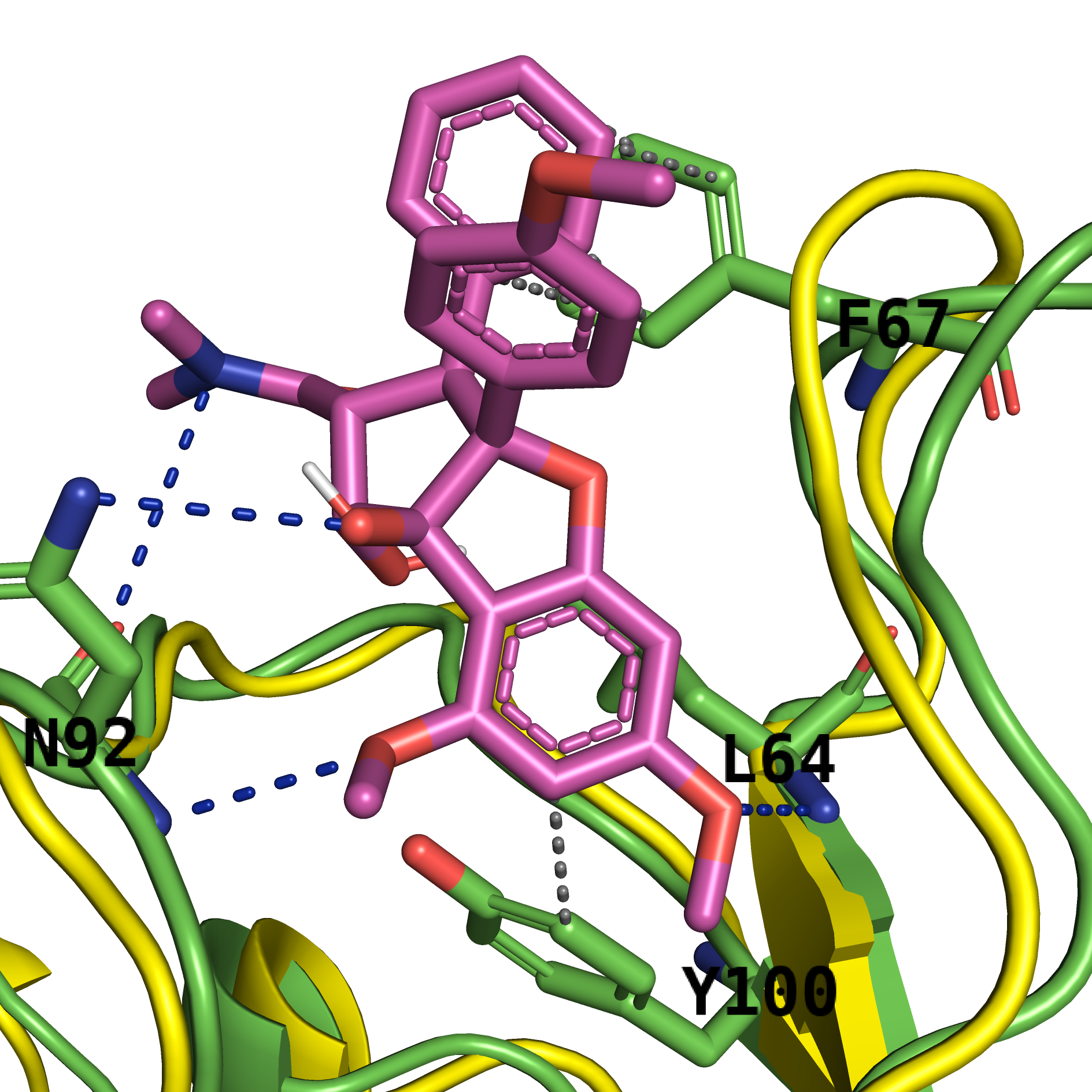

Supplement: Supplementary file 1 [file molecules-30-00840-s001.zip › Figure 5 raw data/44-16.png]

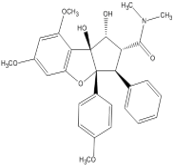

Supplement: Supplementary file 1 [file molecules-30-00840-s001.zip › Figure 5 raw data/E.tif]

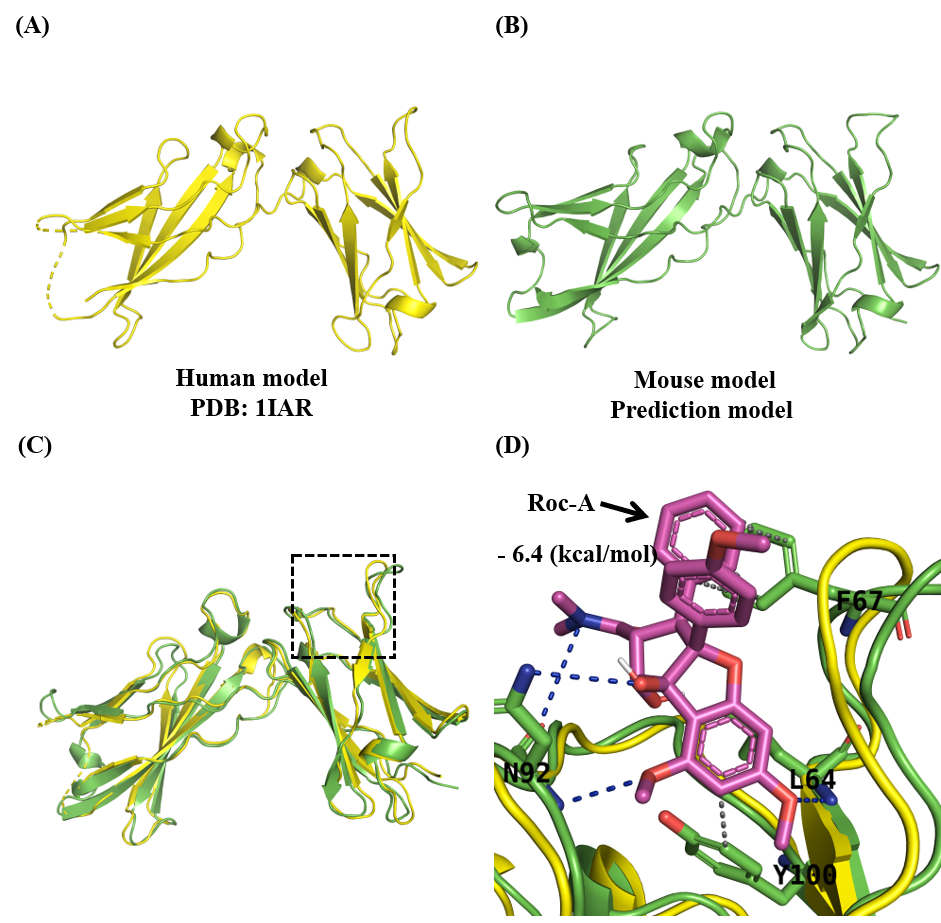

Supplement: Supplementary file 1 [file molecules-30-00840-s001.zip › Figure 5 raw data/figure_5.tif]

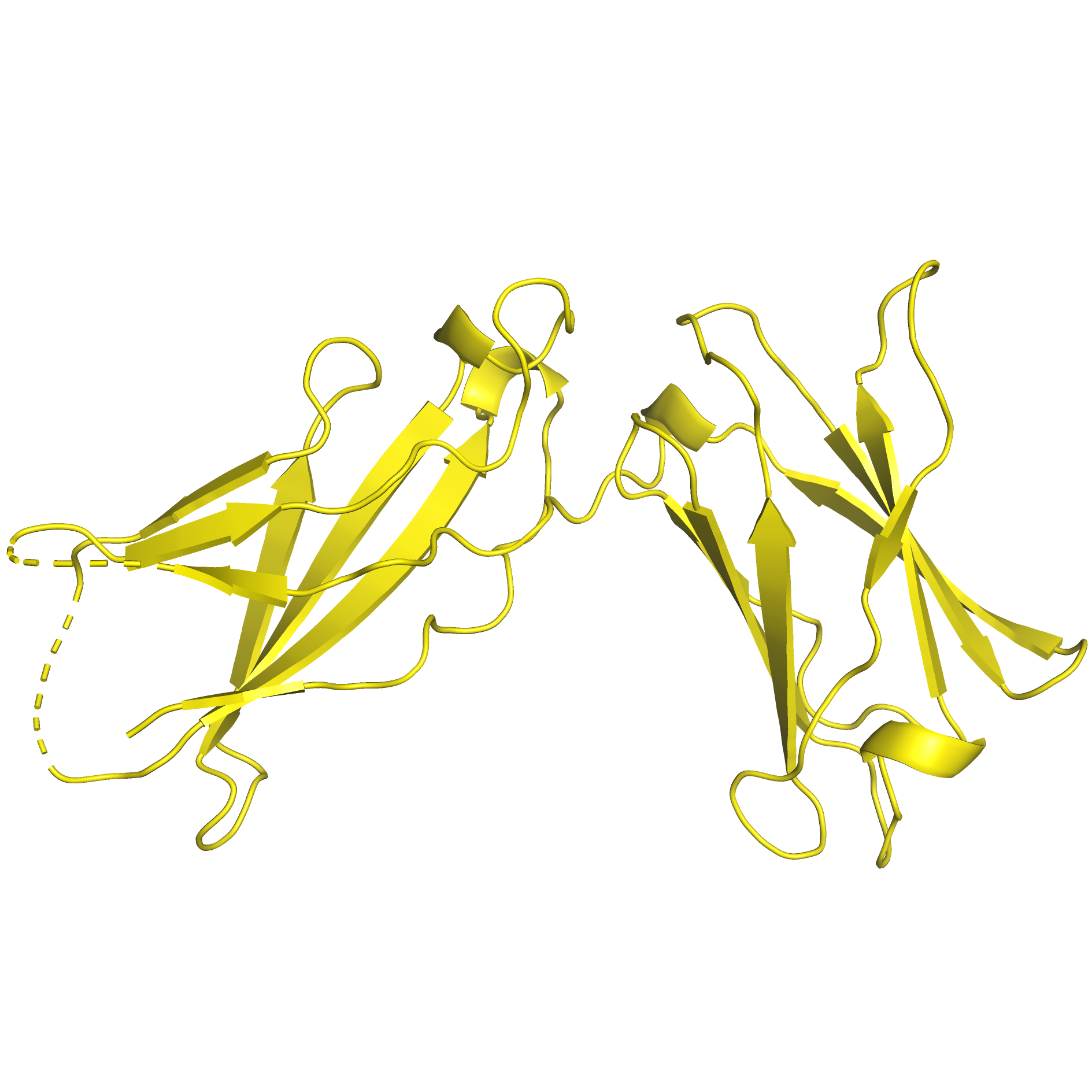

Supplement: Supplementary file 1 [file molecules-30-00840-s001.zip › Figure 5 raw data/human16.png]

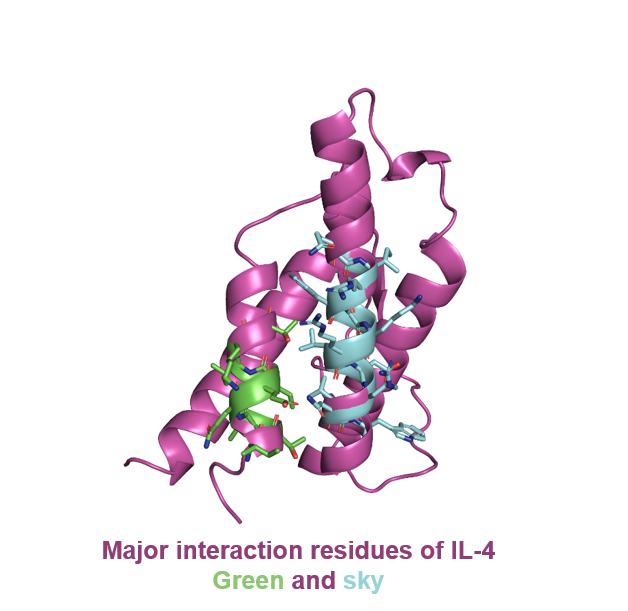

Supplement: Supplementary file 1 [file molecules-30-00840-s001.zip › Figure 5 raw data/IL-4_residues.tif]

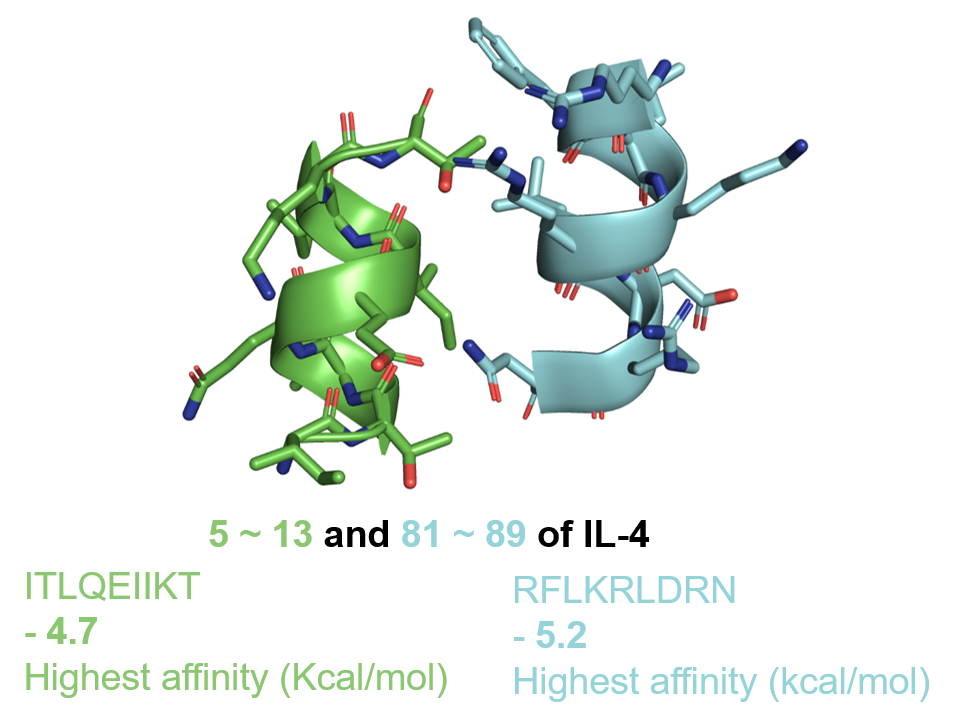

Supplement: Supplementary file 1 [file molecules-30-00840-s001.zip › Figure 5 raw data/IL-4_residues_extraction_from_IL-4.tif]

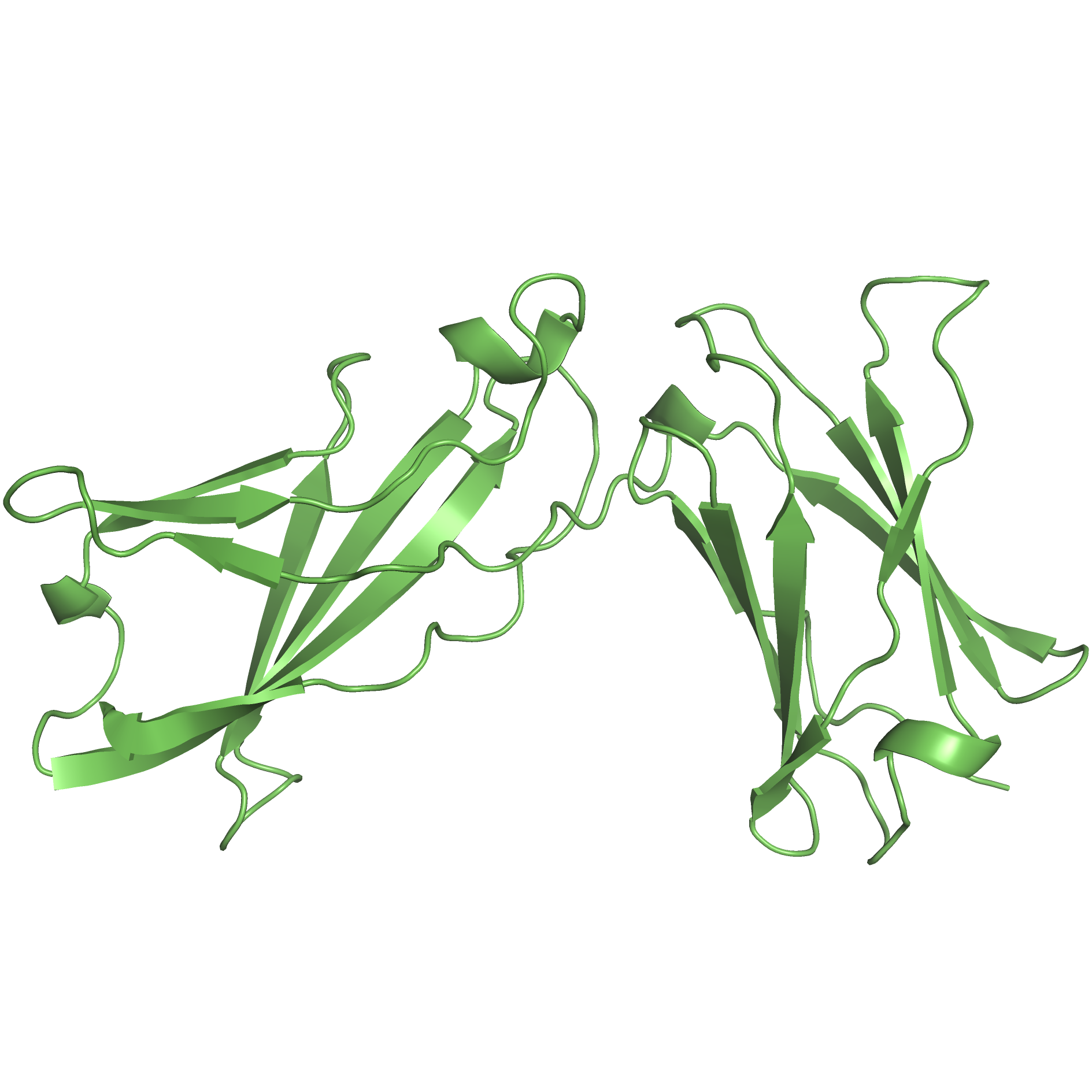

Supplement: Supplementary file 1 [file molecules-30-00840-s001.zip › Figure 5 raw data/mouse16.png]

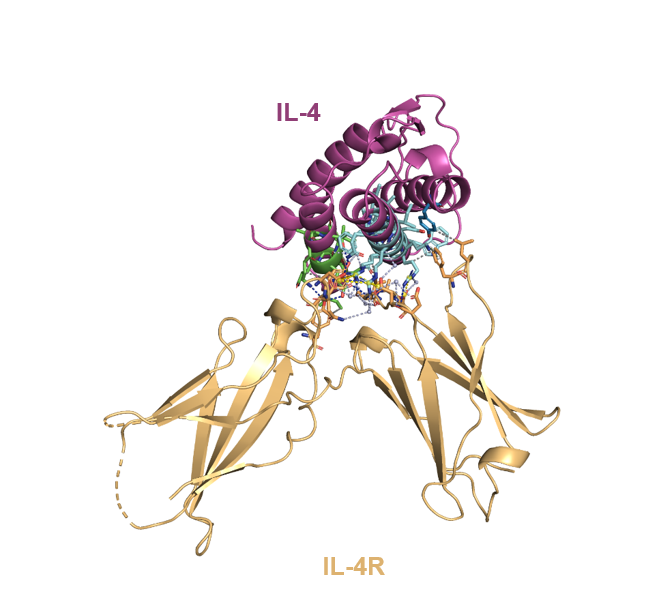

Supplement: Supplementary file 1 [file molecules-30-00840-s001.zip › Figure 5 raw data/real_interaction_structure.tif]

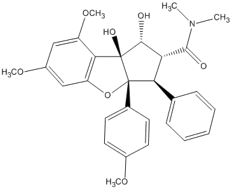

Supplement: Supplementary file 1 [file molecules-30-00840-s001.zip › Figure 5 raw data/roca.tif]

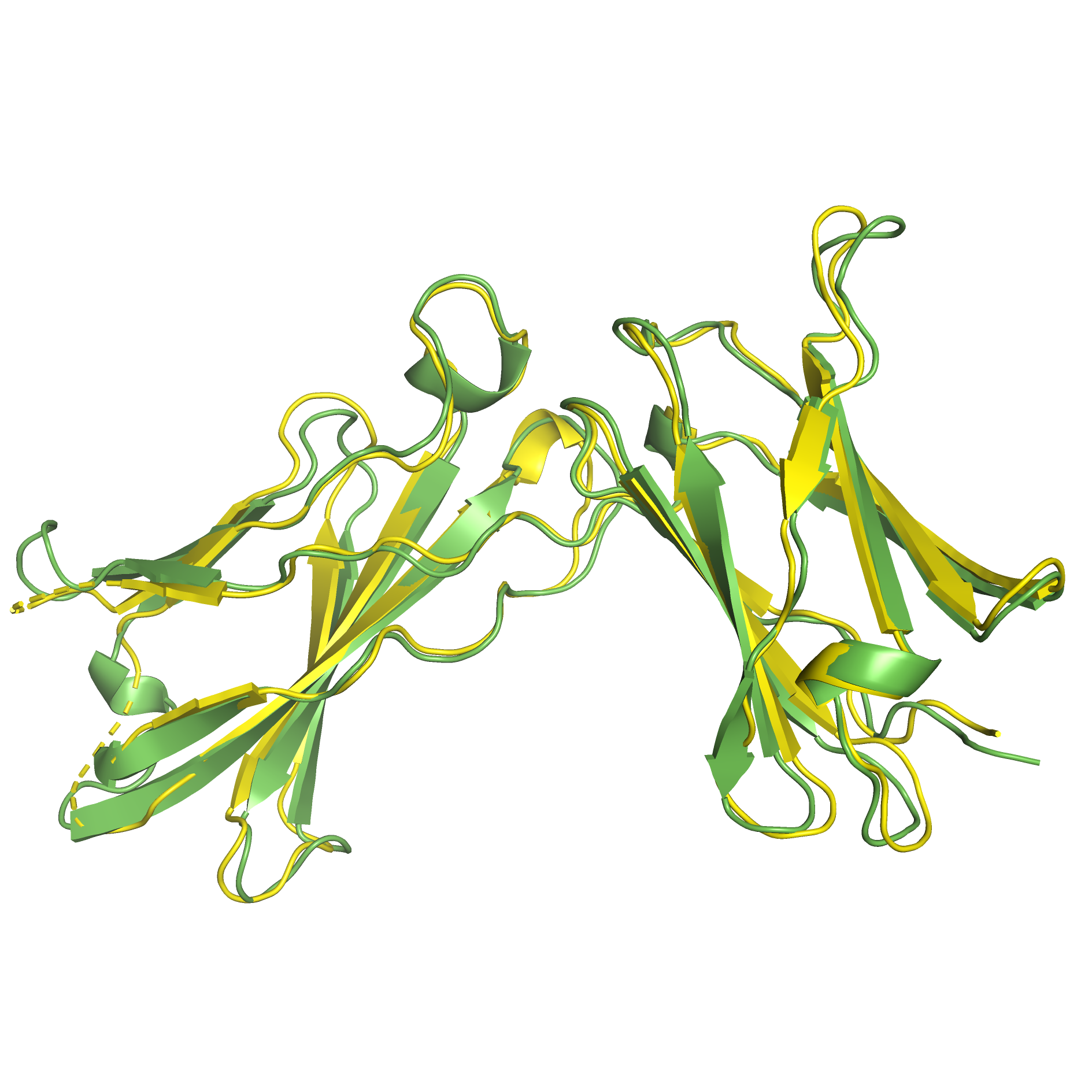

Supplement: Supplementary file 1 [file molecules-30-00840-s001.zip › Figure 5 raw data/superiomposed.png]

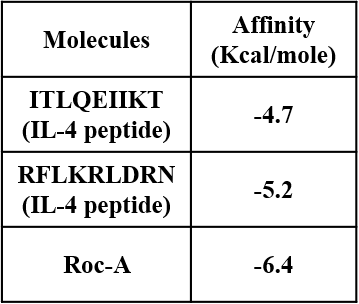

Supplement: Supplementary file 1 [file molecules-30-00840-s001.zip › Figure 5 raw data/Table.png]
